# Dental pulp stem cell exosomes promote angiogenesis via the PI3K/Akt signaling pathway to treat androgenetic alopecia

**DOI:** 10.1186/s13287-026-05056-8

**Published:** 2026-05-21

**Authors:** Wangyu Luo, Yue Shen, Wanning Yu, Le Zhao, Miaomiao Wu, Lihua Xu, Ziyan Liu, Liu Yang, Xiaolei Zhang

**Affiliations:** Department of Stomatology, the Eighth Affiliated Hospital, Sun Yat-sen University, 3025 Shennan Middle Road, Shenzhen, 518033 Guangdong China

**Keywords:** Dental pulp stem cells, Exosomes, Androgenetic alopecia, Angiogenesis

## Abstract

**Background:**

Androgenetic alopecia (AGA), the most common form of hair loss, is currently treated with pharmacological agents that often yield inconsistent results and side effects, highlighting the need for novel therapeutic approaches. This study aimed to investigate the therapeutic potential and molecular mechanisms of exosomes derived from dental pulp mesenchymal stem cells (DPSC-Exos) for AGA.

**Methods:**

We employed a dihydrotestosterone (DHT)-induced AGA mouse model and treated groups with DPSC-Exos or minoxidil. Hair growth was assessed macroscopically and histologically. In vitro, human dermal papilla cells (DPCs) were treated with DHT and/or DPSC-Exos, with or without the PI3K inhibitor LY294002. Analyses included RNA sequencing, RT-qPCR, western blotting, immunofluorescence, and functional assays (proliferation, migration). Statistical significance was determined using Student’s t-test or one-way ANOVA with appropriate post-hoc tests.

**Results:**

DPSC-Exos significantly promoted hair regrowth, increased hair follicle density, and enhanced dermal thickness in mice, with efficacy comparable to minoxidil. Transcriptomic and protein analysis revealed DPSC-Exos activated the PI3K/Akt pathway and upregulated VEGFA, leading to perifollicular vascular network reconstruction. In vitro, DPSC-Exos rescued DHT-induced suppression of DPC proliferation, migration, and expression of hair-inductive markers (ALP, α-SMA). These effects were mediated through upregulation of pro-angiogenic factors (VEGFA, FGF2, ANGPT1) and were completely abolished by PI3K inhibition, confirming the pathway’s necessity. A positive feedback loop between PI3K/Akt activation and VEGFA expression was identified.

**Conclusion:**

Our findings demonstrate that DPSC-Exos promote hair regeneration by activating the PI3K/Akt-VEGFA axis, thereby restoring the follicular vascular niche and DPC function. This study positions DPSC-Exos as a promising, cell-free therapeutic strategy for AGA, with clear mechanistic foundations for future translational development.

**Supplementary Information:**

The online version contains supplementary material available at https://doi.org/10.1186/s13287-026-05056-8.

## Introduction

Androgenetic alopecia (AGA) represents a widespread condition globally, with prevalence rates reaching approximately 80% in men and 40% in women [1]. AGA is a polygenic disorder with hereditary predisposition, in which androgens play a decisive role in its pathogenesis. Testosterone is converted to dihydrotestosterone (DHT) by 5α-reductase, and DHT can suppress hair growth through androgen receptor (AR)-mediated signaling in hair follicles [2, 3]. Other factors such as genetics, psychological stress, poor lifestyle habits, and excessive sun exposure can also exacerbate AGA symptoms [4]. Current clinical options primarily include topical minoxidil, oral finasteride, and hair transplantation [5]. However, pharmacological interventions are often associated with drawbacks such as recurrence and severe cutaneous reactions, while the suitability and success rate of hair transplantation remain contentious [6]. Therefore, identifying novel and potential therapeutic approaches for AGA patients is an urgent clinical need.

Mesenchymal stem cell-derived exosomes (MSC-Exos), which are lipid bilayer-enclosed vesicles measuring 30–200 nm across, have recently attracted growing interest due to their encouraging outcomes in hair regeneration studies and their broad translational prospects [7, 8]. They mediate intercellular communication by direct membrane fusion, endocytic uptake, or ligand-receptor interactions, thereby contributing to diverse physiological and pathological processes, including immune responses, antigen presentation, cell migration and differentiation, as well as tumor invasion [9]. Studies have shown that MSC-Exos are enriched with growth factors and bioactive molecules, enabling them to penetrate deep into damaged tissues, promote cell regeneration and collagen synthesis, and accelerate tissue repair [10]. In recent years, exosomes have gained increasing attention in dermatological fields, including anti-aging, wound healing, skin oncology, and hair regeneration [11, 12].

Notably, tooth development and hair follicle morphogenesis share conserved features, particularly in epithelial–mesenchymal interactions [13]. DPSCs have also been shown to possess regenerative potential for hair follicle formation [14]. Studies have shown that conditioned medium from dental pulp stem cells can effectively ameliorate chemically induced alopecia [15]. Moreover, DPSC-Exos exhibit favorable pro-angiogenic properties and accelerate cutaneous wound healing [16]. Based on these findings, this study focuses on exploring the hair-inductive potential and underlying mechanisms of DPSC-Exos, aiming to provide a translationally relevant, cell-free therapeutic strategy for AGA.

## Methods and materials

Cell.

### Human dermal papilla cells (hDPCs) culture

Human dermal papilla cells (hDPCs; iCell, Shanghai, China) were cultured in DMEM/F12 medium (Gibco, USA) supplemented with 1% penicillin/streptomycin (Gibco, USA), 10% fetal bovine serum (FBS; Gibco, USA), and 100 ng/mL fibroblast growth factor (Sigma, USA). The cells were maintained in an incubator at 37 °C with 5% CO_2_. Cells at passages 3 to 5 (P3–P5) were used for the experiments.

### Isolation, culture, and characterization of human dental pulp stem cells (DPSCs)

DPSCs were obtained from healthy third molars collected from donors aged 18–25 years. All procedures were conducted in accordance with the approved protocols of the Ethics Committee of The Eighth Affiliated Hospital of Sun Yat-sen University (Approval No. 2025‑058‑01).

Pulp tissues were extracted from freshly extracted teeth, minced, and finely digested in 3 mg/mL type I collagenase (Biofroxx, German) at 37 °C for 30 min. The digested tissue was centrifuged at 1000 rpm for 3 min, and the pellet was seeded into T25 flasks (Biofil, Guangzhou, China). Primary cells were cultured in α-MEM (Gibco, USA) with 20% FBS and 2% penicillin/streptomycin. Following passaging, cells were routinely maintained at 37 °C in a 5% CO_2_ atmosphere. The growth medium consisted of α-MEM (Gibco, USA), supplemented by 10% FBS (Gibco, USA) and 1% penicillin/streptomycin (Gibco, USA).

For differentiation, P3 DPSCs were induced into osteogenic and adipogenic lineages for 21 days and stained with Alizarin Red S (Cyagen, USA) and Oil Red O (Cyagen, USA), respectively. Flow cytometry was employed to characterize cell surface markers. The analysis was performed following the protocol provided with the BD Stemflow™ Human MSC Analysis Kit (BD Biosciences, USA).

### Isolation and characterization of DPSC-derived exosomes (DPSC-Exos)

DPSCs from passages 3–5 (P3–P5) were used. After achieving 70–80% confluency, they were subjected to a 24-hour incubation in serum-free α-MEM. The collected conditioned medium was subjected to sequential centrifugation to remove cells and debris: first at 300 × g for 10 min, then at 3000 × g for 15 min, with both steps performed at 4 °C. The supernatant was concentrated using a 100 kDa ultrafilter (Millipore, USA) at 4000 × g for 15 min. Exosomes were precipitated by adding Total Exosome Isolation Reagent (Thermo Fisher, USA) at a 2:1 ratio (medium: reagent), incubating overnight at 4 °C, and centrifuging at 10,000 × g for 1 h. For long-term storage, the pellet was resuspended in PBS and placed at − 80 °C.

A multi-method approach was conducted for exosome characterization: size distribution analysis was performed via nanoparticle tracking analysis (NTA); morphological examination was carried out using transmission electron microscopy (TEM); and protein marker expression was verified by Western blotting with antibodies (1:500, Zenbio, China) against the positive markers CD81 and TSG101 as well as the negative marker Calnexin.

### Cellular uptake of DPSC-Exos

DPSC-Exos (100 µg) were labeled with the green fluorescent dye Dio (10 µM; Beyotime, Shanghai, China) and re-isolated following the aforementioned method. The labeled exosomes were incubated with DPCs for 6 h at 37 °C. After being fixed with 4% paraformaldehyde for 10 min and stained with phalloidin (100 µM, Glpbio, USA) for 30 min, the cells were mounted. An antifade mounting medium containing DAPI (Beyotime, China) was applied prior to sealing the coverslips. Confocal imaging was performed on a Zeiss LSM 880 microscope for subsequent analysis. All procedures were performed protected from light.

### Cell proliferation assay

To evaluate cell proliferation, the CCK-8 assay (Fdbio, Hangzhou, China) was employed. Briefly, DPCs were plated in 96-well plates at a density of 5 × 10³ cells per well and treated with DPSC-Exos at concentrations of 0, 5, 10, and 20 µg/mL, respectively. At designated time points (24, 48, and 72 h), the culture supernatant was first carefully removed from each well. Subsequently, 100 µL of CCK-8 working solution (diluted to 10% in medium) was added. After an additional 1-hour incubation, the absorbance at 450 nm was recorded using a microplate reader.

### Cell migration assay

A scratch assay was performed to evaluate the cell migration ability. DPCs (2 × 10⁵ cells per well) were seeded in 6-well platesand cultured until 90–100% confluent. A uniform wound was created in each well using a 200 µL sterilized pipette tip. After washing with PBS, the cells were treated with serum-free medium either alone or containing 20 µg/mL DPSC-Exos. Images of the wound areas were captured at 0, 24 h post-scratching.

### Quantitative real-time PCR (RT-qPCR)

Total RNA was extracted from DPCs using a commercial RNA extraction kit (Goonine, Guangzhou, China) according to the manufacturer’s instructions. cDNA was synthesized by reverse transcription. Quantitative PCR was performed using SYBR Green dye (AGBIO, Hunan, China). The primer sequences used are listed in the Table 1.

**Table 1:** Primers for RT-qPCR

Primers		Sequence (5’-3’)
*GAPDH*	Forward	GCACCGTCAAGGCTGAGAAC
	Reverse	TGGTGAAGACGCCAGTGGA
*ALP*	Forward	AACATCAGGGACATTGACGTG
	Reverse	GTATCTCGGTTTGAAGCTCTTCC
*α-SMA*	Forward	AAGATCAAGATCATCGCCCCG
	Reverse	CCTCGTCGTACTCCTGCTTG
*VEGFA*	Forward	CGAAACCATGAACTTTCTGC
	Reverse	CCTGAGTGGGCACACACTCC
*FGF2*	Forward	AGCGACCCTCACATCAAG
	Reverse	ATCTTCCATCTTCCTTCATAGC
*ANGTP1*	Forward	CATTCTTCGCTGCCATTCTG
	Reverse	GCACATTGCCCATGTTGAATC

### **Immunofluorescence staining**

Cells were fixed with 4% paraformaldehyde (Biosharp, Beijing, China) for 20 min, permeabilized with 0.25% Triton X-100 (Solarbio, Beijing, China) for 10 min, and blocked with 5% bovine serum albumin (BSA; Solarbio, Beijing, China) for 30 min at room temperature. Samples were incubated overnight at 4 °C with primary antibodies against Ki67 (rabbit, 1:200; abcam, USA) and α-SMA (rabbit, 1:100; HUABIO, Hangzhou, China). After washing, cells were incubated with species-matched fluorescent secondary antibodies (Alexa Fluor 594; CST, USA) for 1 h at room temperature in the dark. Nuclei were then counterstained with DAPI (Beyotime, Shanghai, China). Images were acquired using a Zeiss inverted fluorescence microscope and analyzed with Image J software.

### Western blotting

Cells were collected and lysed using RIPA buffer (Fdbio, Hangzhou, China) supplemented with protease and phosphatase inhibitor cocktails (MCE, USA). The protein concentration of the lysates was determined using a BCA assay (Absin, Shanghai, China). Subsequently, protein samples were mixed with 5× loading buffer (Fdbio, Hangzhou, China) and denatured by heating at 95 °C for 5 min. Equal amounts of protein (20 µg per lane) were separated by SDS-PAGE (Epizyme, Shanghai, China) and then transferred onto a 0.45 μm PVDF membrane (Millipore, USA). The membrane was blocked with 5% non-fat milk (Servicebio, Wuhan, China) for 1 h at room temperature, followed by incubation with primary antibodies overnight at 4 °C. Detailed information on the antibodies used is provided in Table 2. After washing with TBST, the membrane was incubated with HRP-conjugated secondary antibody for 1 h at room temperature. Protein bands were visualized using enhanced chemiluminescence (ECL; Fdbio, Hangzhou, China) and imaged with a Bio-Rad ChemiDoc™ system. Densitometric analysis was performed using ImageJ.

**Table 2:** List of primary antibodies used for Western blot

Antibody Target	Dilution	Host Species	Company
GAPDH	1:5000	Rabbit	CST, USA
ALP	1:2000	Rabbit	HUABIO, Hangzhou, China
ALP	1:1000	Rabbit	Proteintech, Wuhan, China
α-SMA	1:10000	Rabbit	HUABIO, Hangzhou, China
VEGFA	1:1000	Rabbit	Proteintech, Wuhan, China
p-PI3K	1:1000	Rabbit	Abcam, USA
PI3K	1:1000	Rabbit	CST, USA
p-AKT	1:2000	Rabbit	HUABIO, Hangzhou, China
AKT	1:5000	Rabbit	HUABIO, Hangzhou, China

#### Animal

### In vivo hair regrowth model and treatment

Twenty-four male C57BL/6 mice (6 weeks old) were obtained from Bai Shi Tong. The study was approved by the Ethics Committee of The Eighth Affiliated Hospital of Sun Yat-sen University (Approval No. 2025-020-01). After 7 days of acclimatization, a dorsal area of 2 cm × 3 cm was shaved on each mouse. The pink skin color confirmed that all hair follicles were in the telogen phase. 24 mice were randomly divided into four groups (*n* = 6 per group): PBS (negative control), DHT (1 mg/day DHT, AGA model group), DHT + Exos (1 mg/d DHT + 50 µg/day DPSC-Exos, treatment group), and DHT + minoxidil (1 mg/d DHT + 5% minoxidil, positive control). DPSC-Exos were administered by daily subcutaneous injection (total 50 µg/day per mouse) at six evenly distributed sites, whereas 5% minoxidil was applied topically to the shaved dorsal skin once daily. Hair regrowth was assessed using a predefined scoring system based on photographs taken on days 1, 6, 11, 13, and 15. Hair regrowth was then evaluated by three independent investigators under blinded conditions using a predefined scoring system [17].

For hair growth observation and photography, mice were anesthetized by inhalation of isoflurane (RWD, Shenzhen, China; induction: 3–5%; maintenance: 1–2%). At the end of the experiments on day 16, all mice were euthanized by cervical dislocation under deep anesthesia with isoflurane. Every effort was made to minimize animal suffering.

### Histological analysis and immunostaining of skin tissues

On Day 16, the depilated dorsal skin tissues were harvested and fixed in 10% formalin (Biosharp, Beijing, China), followed by paraffin embedding and sectioning (4 μm thickness). Hematoxylin and eosin (HE; Solarbio, Beijing, China) staining was performed to quantitatively analyze hair follicle’s number and dermal thickness. Sections were baked at 65 °C for 1 h, followed by deparaffinization and rehydration through a graded series of xylene and ethanol (100%, 95%, 85%, and 75%). Antigen retrieval was conducted using citrate buffer (Servicebio, Wuhan, China). Endogenous peroxidase activity was blocked with 3% hydrogen peroxide for 10 min, and nonspecific binding was reduced by incubation with 10% goat serum (Zsbio, Beijing, China) for 30 min at room temperature. The sections were then incubated overnight at 4 °C with primary antibodies against anti-Ki67 (rabbit, 1:200; Abcam, USA), anti-α-SMA (rabbit, 1:100; HUABIO, Hangzhou, China) and AR (rabbit, 1:200; Abcam, USA). After washing with PBS, sections were incubated for 1 h at room temperature with either an Alexa Fluor 594-conjugated goat anti-rabbit IgG for immunofluorescence or an HRP-conjugated goat anti-rabbit IgG for immunohistochemical. For immunofluorescence, nuclei were counterstained with DAPI(Beyotime, Shanghai, China); for immunohistochemical, color development was performed using DAB (Zsbio, Beijing, China). All sections were mounted and observed under a Zeiss inverted microscope. Quantitative or semi-quantitative analysis was carried out using ImageJ software.

### Western blotting of skin tissues

Total protein was extracted from skin tissues as follows. Approximately 20 mg of tissue was rinsed with ice-cold PBS, minced, and homogenized on ice in 400 µL of RIPA lysis buffer (Fdbio, Hangzhou, China) containing protease and phosphatase inhibitors (MCE, USA) using a tissue homogenizer. The homogenate was then lysed on a shaking platform at 4 °C for 30 min, followed by centrifugation at 12,000 × g for 15 min at 4 °C. The supernatant was collected as the total protein extract. Protein concentration was determined using a BCA assay (Absin, Shanghai, China). Subsequent sample preparation was performed as described in Sect.  2.9.

### Transcriptome sequencing and bioinformatics analysis

Dorsal skin tissues were collected from the four in vivo groups (PBS, DHT, DHT + Exos, and DHT + minoxidil), with three biological replicates per group. Total RNA was isolated from all samples with Trizol reagent, and its integrity was subsequently evaluated on an Agilent 2100 Bioanalyzer. Following this, sequencing libraries were prepared and subjected to sequencing using the Illumina NovaSeq 6000 platform. The DESeq2 package was then employed to identify differentially expressed genes (DEGs), applying thresholds of |log2(Fold Change)| > 1 and an adjusted p-value < 0.05. Finally, functional enrichment analysis for the identified DEGs was conducted utilizing the Gene Ontology (GO) and Kyoto Encyclopedia of Genes and Genomes (KEGG) databases.

### Statistical analysis

All quantitative data were expressed as mean ± standard error of the mean (SEM). Statistical analyses were performed using GraphPad Prism version 10.0. Comparisons between two groups were analyzed by unpaired Student’s t-test. One-way ANOVA was applied for multiple group comparisons. *p* < 0.05 was considered statistically significant. Each experiment was repeated independently at least three times.

## Results

### Characterization of DPSCs and DPSC-Exos

**Fig. 1:**
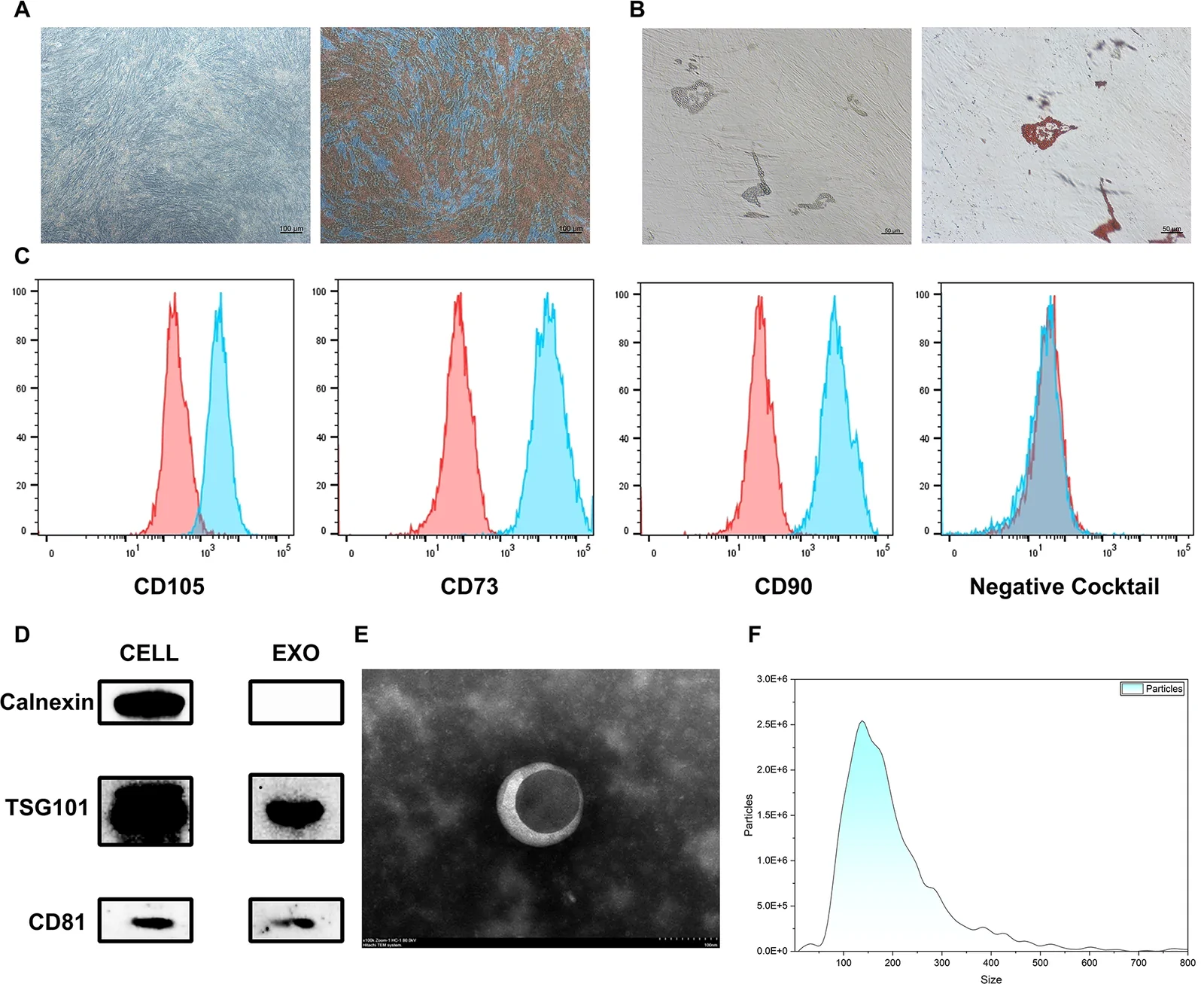
Characterization of dental pulp stem cells (DPSCs) and DPSC-derived exosomes (DPSC-Exos). **A** Alizarin Red S staining of DPSCs after 21 days of osteogenic induction (right) and the corresponding unstained control (left). Scale bar = 100 μm. **B** Oil Red O staining of DPSCs after 21 days of adipogenic induction (right) and the corresponding control (left). Scale bar = 100 μm. **C** Flow cytometric analysis of surface markers expressed on DPSCs. **D** Western blot analysis of exosomal positive markers (CD81 and TSG101) and negative marker (Calnexin) in DPSC-Exos. **E** Representative transmission electron microscopy image showing the cup-shaped morphology of DPSC-Exos. Scale bar = 100 nm. **F** Nanoparticle tracking analysis of DPSC-Exos showing size distribution, with a median particle diameter of 171 nm

DPSCs were isolated from healthy, caries-free third molars extracted from patients aged 18–25 years using enzymatic digestion. Multilineage differentiation assays confirmed the osteogenic and adipogenic potential of DPSCs, as evidenced by Alizarin Red S staining (Fig. 1A) and Oil Red O staining (Fig. 1B), respectively. Flow cytometric analysis revealed that the cells were highly positive for mesenchymal stem cell surface markers CD105 (97.4%), CD73 (99.2%), and CD90 (99.4%), while expression of the hematopoietic/immune lineage marker cocktail (CD34/CD45/CD11b/CD19/HLA-DR) was minimal (0.13%) (Fig. 1C).

DPSC-Exos were isolated from conditioned medium of DPSCs. Western blotting analysis showed that DPSC-Exos expressed the exosomal positive markers TSG101 and CD81, and the absence the negative marker Calnexin (Fig. 1D). Transmission electron microscopy revealed the characteristic cup-shaped, double-membrane vesicular structure of exosomes (Fig. 1E). Nanoparticle tracking analysis indicated that the majority of particles ranged from 100 to 200 nm in diameter, with an average size of 171 nm (Fig. 1F). Collectively, these results confirm the successful isolation and characterization of DPSC-derived exosomes.

### DPSC-Exos promote the proliferation and migration of DPCs in a dose-dependent manner

**Fig. 2:**
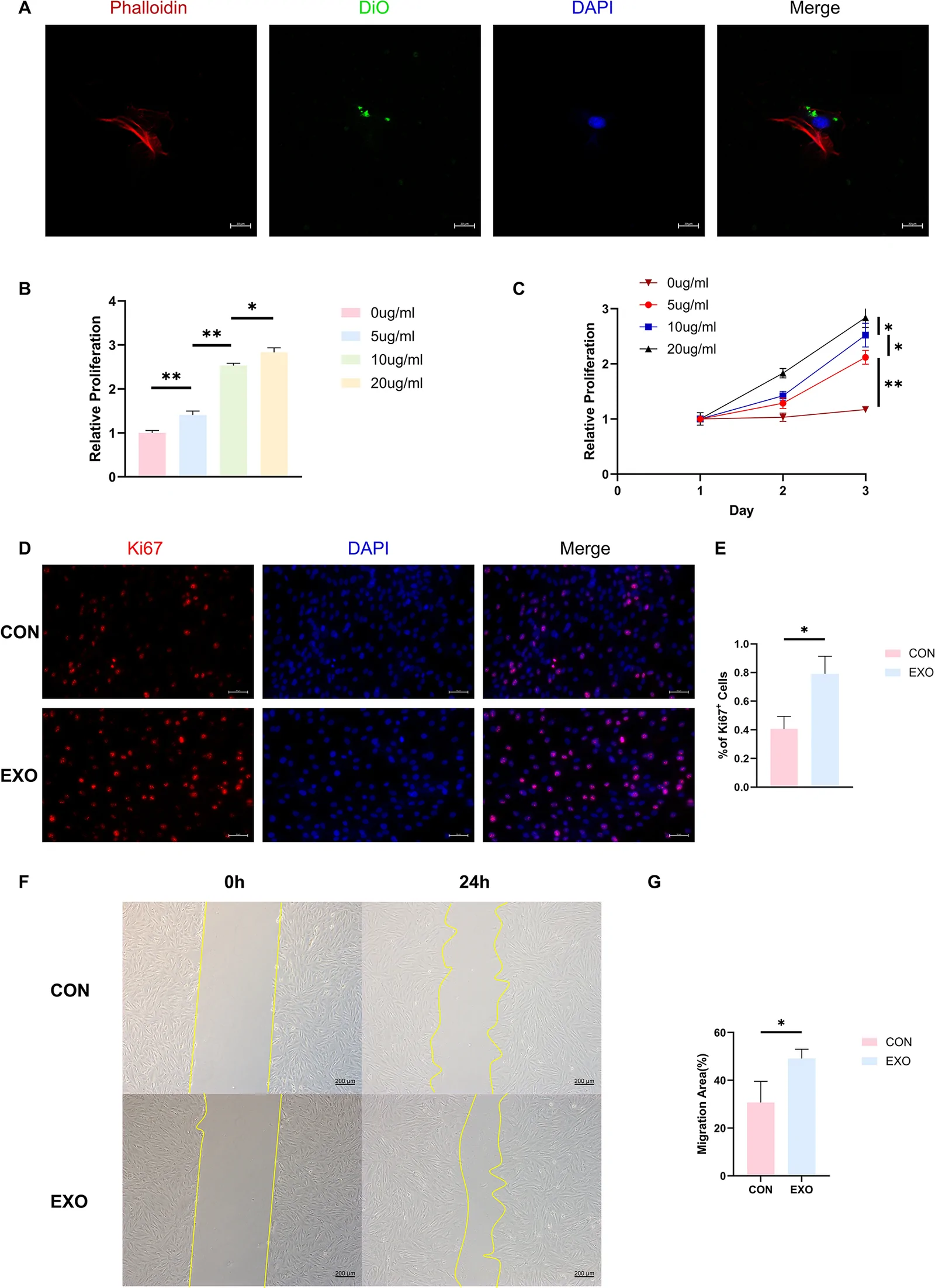
DPSC-Exos promote the proliferation and migration of dermal papilla cells (DPCs). **A** Cellular uptake of DPSC-Exos by DPCs after 6 h of incubation with DiO-labeled DPSC-Exos (green). DPCs were stained with rhodamine–phalloidin (red) and DAPI (blue). Scale bar = 20 μm. **B** Cell viability of DPCs treated with different concentrations of DPSC-Exos (0, 5, 10, 20 µg/mL) for 24 h, as measured by CCK-8 assay. ns, not significant; *P < 0.05, **P < 0.01 vs. 0 µg/mL group. **C** Time- and concentration-dependent effects of DPSC-Exos on DPC proliferation over 1, 2, and 3 days. ns, not significant; *P < 0.05, **P < 0.01 vs. 0 µg/mL group at the corresponding time point. **D** Representative immunofluorescence images of DPCs stained for Ki67 (red) and DAPI (blue) after treatment with or without 20 µg/mL DPSC-Exos. Scale bar = 20 μm. **E** Quantitative analysis of Ki67-positive cells. ns, not significant; *P < 0.05, **P < 0.01. **F** Representative images of scratch-wound assays performed on DPC monolayers treated with or without 20 µg/mL DPSC-Exos for 0 and 24 h. Scale bar = 200 μm. **G** Quantitative analysis of cell migration area. ns, not significant; *P < 0.05, **P < 0.01

Dermal papilla cells (DPCs) are key mesenchymal regulators of hair follicle morphogenesis and cycling, largely through paracrine production of growth factors and cytokines that influence hair follicle stem cell activation and differentiation23. To examine whether DPSC-Exos modulate DPC function, we first assessed cellular uptake. After 6 h of co-culture with DiO-labeled DPSC-Exos, fluorescent signals were readily detected within DPCs, indicating efficient internalization (Fig. 2A).

When DPCs were treated with different concentrations of DPSC-Exos (0 µg/ml, 5 µg/ml, 10 µg/ml, 20 µg/ml), a significant and concentration-dependent increase in cell proliferation was observed compared with the control group (Fig. 2B, C). Ki67 immunofluorescence staining further confirmed the pro-proliferative effect of 20 µg/ml DPSC-Exos on DPCs (Fig. 2D, E). In addition, a scratch-wound assay demonstrated that DPSC-Exos also promoted the migration of DPCs (Fig. 2F, G).

### DPSC-Exos upregulate hair-inductive markers in DPCs

**Fig. 3:**
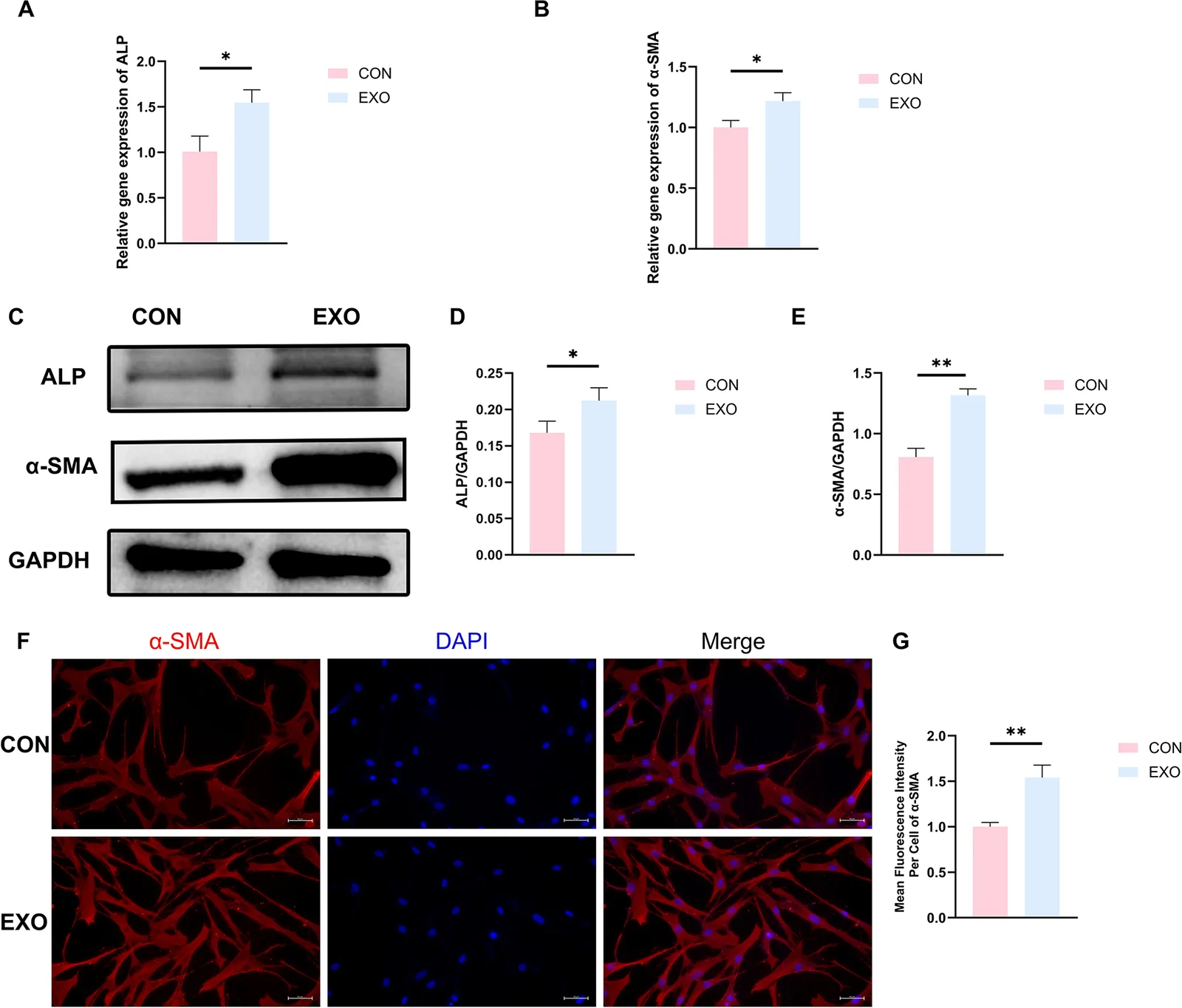
DPSC-Exos upregulate hair-inductive markers in DPCs. **A**, **B** RT-qPCR analysis of ALP (A) and α-SMA (B) mRNA levels in DPCs treated with or without DPSC-Exos. Gene expression was normalized to GAPDH. ns, not significant; *P < 0.05, **P < 0.01. **C** Representative Western blot images showing ALP and α-SMA protein expression in DPCs. **D**, **E** Densitometric quantification of the bands in (C) for ALP (D) and α-SMA (E). ns, not significant; *P < 0.05, **P < 0.01. **F** Representative immunofluorescence images of DPCs stained for α-SMA (red) and DAPI (blue). Scale bar = 50 μm. **G** Quantitative analysis of the mean fluorescence intensity of α-SMA. ns, not significant; *P < 0.05, **P < 0.01

Alkaline phosphatase (ALP) activity and α-smooth muscle actin (α-SMA) expression are commonly used markers associated with the hair-inductive phenotype of DPCs24. To examine whether DPSC-Exos influence this activity, we assessed the expression of ALP and α-SMA using RT-qPCR and Western blotting. Compared with the control group, DPSC-Exo-treated DPCs showed significantly increased mRNA levels of both *ALP* and *α-SMA* (Fig. 3A, B). Consistently, protein expression of ALP and α-SMA was also markedly upregulated after exosome treatment (Fig. 3C–E). Immunofluorescence staining further confirmed increased α-SMA expression in DPSC-Exos–treated cells (Fig. 3F-G), supporting an enhanced hair-inductive state.

### DPSC-Exos reverse the inhibitory effects of DHT on DPCs

**Fig. 4:**
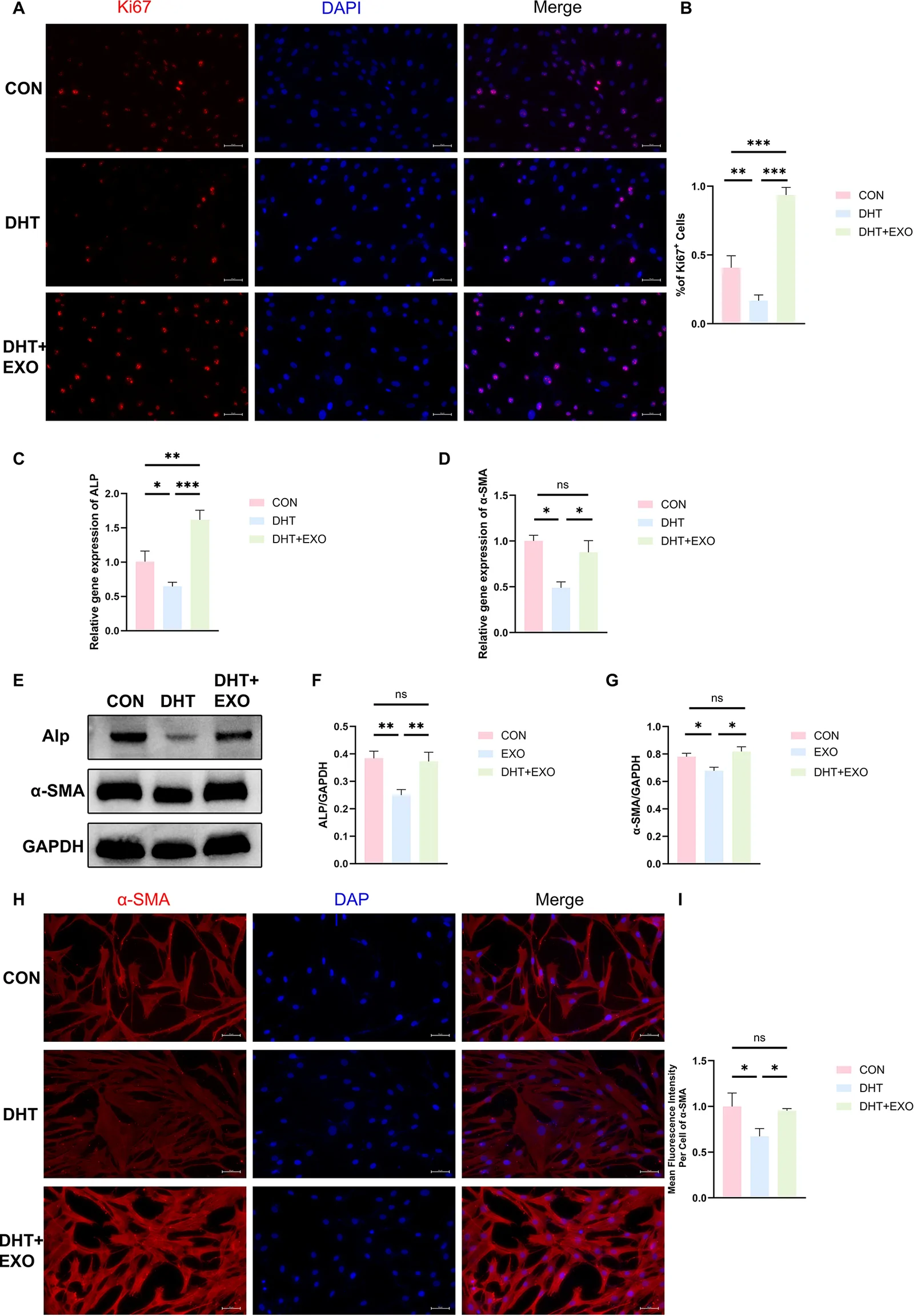
DPSC-Exos counteract the inhibitory effects of DHT on DPCs. **A** Representative immunofluorescence images of Ki67 (red) and Dapi (blue) in DPCs treated with Control, DHT, or DHT + DPSC-Exos. Scale bar = 50 μm. **B** Quantitative analysis of Ki67-positive cells. ns, not significant; *P < 0.05, **P < 0.01, ***P < 0.001. **C**, **D** RT-qPCR analysis of *ALP* (C) and *α-SMA* (D) mRNA levels. Expression was normalized to *GAPDH*. ns, not significant; *P < 0.05, **P < 0.01, ***P<0.001. **E** Representative Western blot images showing ALP and α-SMA protein expression. **F**, **G** Densitometric quantification of ALP (F) and α-SMA (G) protein levels from (E). ns, not significant; *P < 0.05, **P < 0.01. **H** Representative immunofluorescence images of α-SMA (red) and DAPI (blue). Scale bar = 50 μm. **I** Quantitative analysis of the mean fluorescence intensity of α-SMA. ns, not significant; *P < 0.05, **P < 0.01

Having demonstrated that DPSC-Exos promote the hair-inductive capacity and proliferation of DPCs, we next asked whether exosomes could mitigate the suppression induced by DHT (10 µmol/L). First, Ki67 immunofluorescence analysis showed that DHT treatment reduced Ki67 expression in DPCs, whereas co-treatment with DPSC-Exos significantly restored Ki67 levels (Fig. 4A, B). Furthermore, RT-qPCR and Western blot analyses revealed that DHT downregulated both mRNA and protein expression of the hair-inductive markers ALP and α-SMA (Fig. 4C–G), indicating impaired hair-inductive function. Importantly, DPSC-Exos treatment effectively reversed this downregulation, elevating ALP and α-SMA expression to levels comparable to or even exceeding those in the control group. Consistent with these findings, α-SMA immunofluorescence further confirmed that DPSC-Exos alleviated the DHT-induced suppression of DPCs (Fig. 4H, I). These results suggest that DPSC-Exos alleviate DHT-mediated functional impairment of DPCs.

### DPSC-Exos promote hair regrowth in a DHT-induced AGA mouse model

**Fig. 5:**
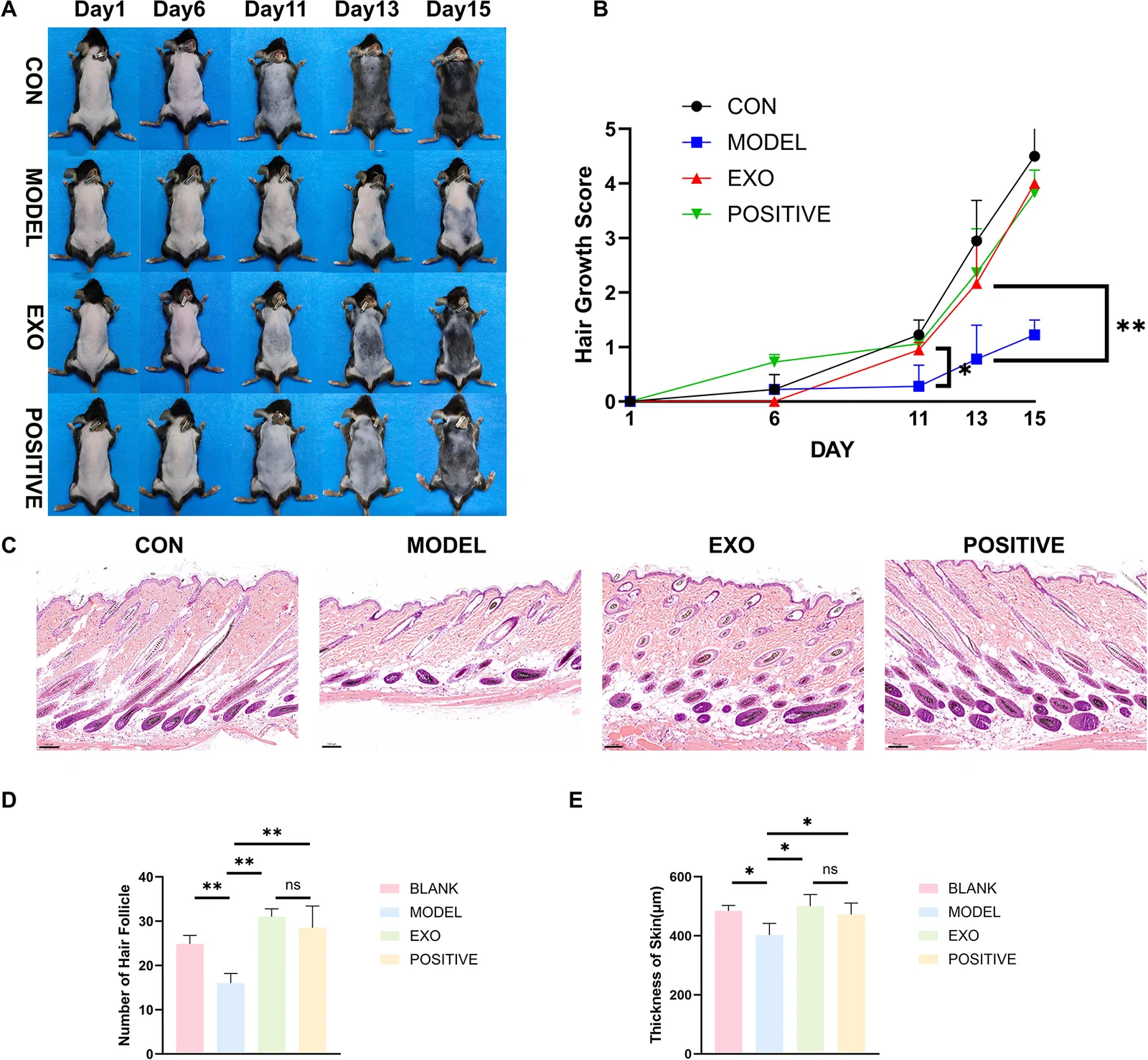
DPSC-Exos promote hair regrowth in a DHT-induced AGA mouse model. **A** Representative dorsal photographs of C57BL/6 mice taken on days 1, 6, 11, 13, and 15 after depilation and treatment. Mice were divided into four groups (*n* = 6 per group): Control (sham), Model (DHT), EXO (DHT + DPSC-Exos), and Positive (DHT + minoxidil). **B** Hair growth scores of each group at the indicated time points. ns, not significant; *P < 0.05, **P < 0.01. **C** H&E staining of dorsal skin sections collected on day 16. Scale bar = 100 μm. **D** Quantitative comparison of hair follicle numbers per field. ns, not significant; *P < 0.05, **P < 0.01. **E** Quantitative comparison of dermal thickness. ns, not significant; *P < 0.05, **P < 0.01

To assess the in vivo effect of DPSC-Exos on hair growth, 24 C57BL/6 mice (6 weeks old) in the telogen phase were selected. A dorsal area of 2 × 3 cm was shaved, exposing pink skin. Mice were randomly divided into four groups (*n* = 6 per group): a sham-treated control group (CON), a model group (MODEL) receiving daily subcutaneous injection of DHT (1 mg/day per mouse), an exosome-treated group (EXO) receiving daily subcutaneous injection of 50 µg DPSC-Exos, and a positive control group (POSITIVE) treated daily with 5% minoxidil. Hair growth was photographed on days 1, 6, 11, 13, and 15 (Fig. 5A) and scored using a hair growth index (Fig. 5B). On day 11, the skin of the EXO and POSITIVE groups had turned gray, indicating entry into the anagen phase, whereas the MODEL group remained pink (telogen phase). By day 13, the EXO group showed visible short black hair, comparable to the POSITIVE group, while the MODEL group had only just begun to turn gray. On day 16, dorsal skin samples were collected and subjected to H&E staining (Fig. 5C). Compared with the MODEL group, the EXO group exhibited a significant increase in both hair follicle number and dermal thickness, reaching levels similar to those in the POSITIVE group (Fig. 5D, E).

### DPSC-Exos attenuate DHT-induced AR upregulation and restore proliferative activity in vivo

**Fig. 6:**
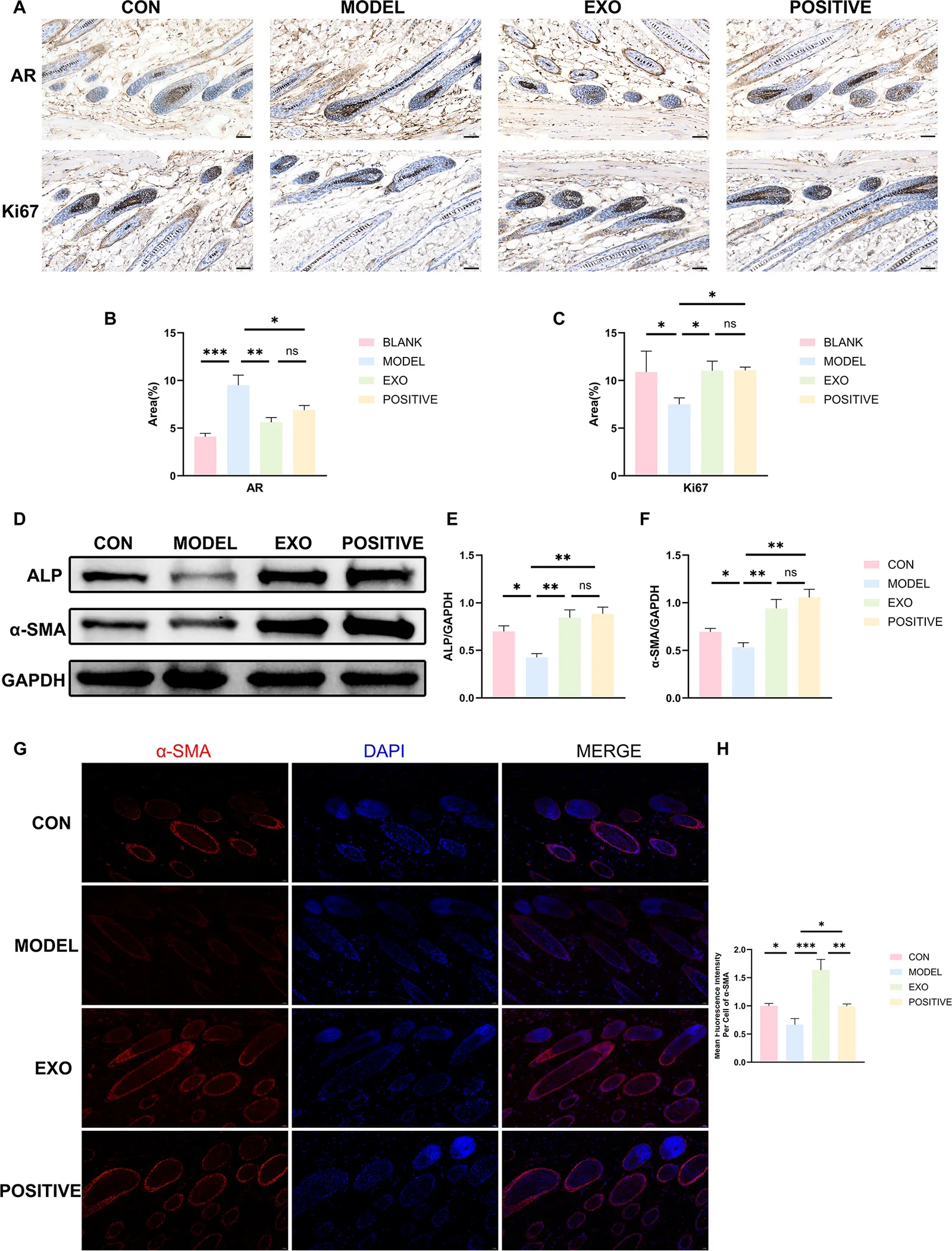
DPSC-Exos attenuate DHT-induced AR upregulation and restore proliferative activity in vivo. **A** Representative immunohistochemical staining of Ar and Ki67 in hair follicles from each group. Scale bar = 50 μm. **B**, **C** Quantitative analysis of the positive area for AR (B) and Ki67 (C). ns, not significant; *P < 0.05, **P < 0.01, ***P<0.001. **D** Representative Western blot images showing ALP and α-SMA protein expression in skin tissues. **E**, **F** Densitometric quantification of ALP (E) and α-SMA (F) protein levels from (D). ns, not significant; *P < 0.05, **P < 0.01. **G** Representative immunofluorescence images of α-SMA (red) and DAPI (blue) in skin sections. Scale bar = 50 μm. **H** Quantitative analysis of the mean fluorescence intensity of α-SMA. ns, not significant; *P < 0.05, **P < 0.01, ***P<0.001

Given that DHT-mediated inhibition of hair regeneration is known to involve the androgen receptor (AR), we performed AR immunohistochemistry on skin sections. AR immunostaining was detectable in hair follicles across groups. The MODEL group showed a significantly larger AR-positive area compared with the CON, EXO, and POSITIVE groups (Fig. 6A, B), indicating that DPSC-Exos alleviated DHT-induced AR overexpression.

In parallel, Ki67 staining demonstrated higher proliferative activity within hair follicles in the CON, EXO, and POSITIVE groups than in the MODEL group (Fig. 6A, C). Furthermore, Western blot analysis revealed that the expression of key anagen-phase markers, ALP and α-SMA, was significantly downregulated in the MODEL group compared with the CON group. Notably, DPSC-Exos treatment effectively restored their expression levels, showing a statistically significant increase relative to the MODEL group (Fig. 6D-F). This restorative effect was further corroborated by α-SMA immunofluorescence staining (Fig. 6G, H). Collectively, DPSC-Exos mitigated DHT-induced hair growth impairment in vivo and exhibited an overall effect comparable to minoxidil under the conditions tested.

### DPSC-Exos promote hair regeneration by activating the PI3K/Akt signaling pathway in vivo

**Fig. 7:**
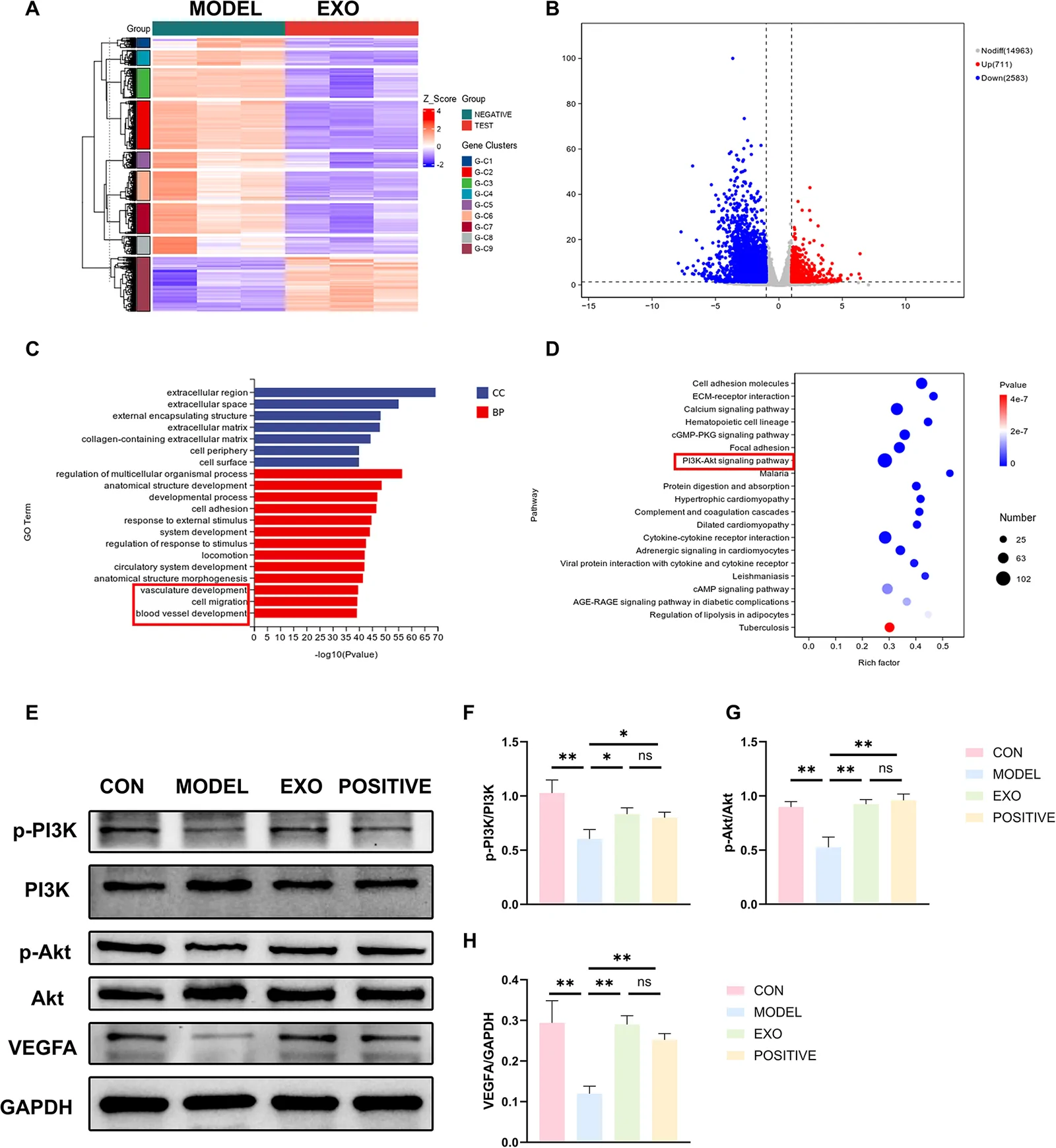
DPSC-Exos promote hair regeneration by activating the Pi3k/Akt signaling pathway in vivo. **A** Heatmap showing differentially expressed genes between the MODEL and EXO groups from transcriptomic sequencing of mouse dorsal skin. **B** Volcano plot illustrating the significance and magnitude of gene expression changes between the MODEL and EXO groups. **C** GO term enrichment analysis of biological processes significantly altered by DPSC-Exos treatment. **D** KEGG pathway enrichment analysis, highlighting the PI3K/Akt signaling pathway as a key upregulated pathway in the EXO group. **E** Representative Western blot images showing the expression levels of p-PI3K, total PI3K, p-Akt, total Akt, and VEGFA in skin tissues from each group. **F–H** Densitometric quantification of protein levels for p-PI3K/PI3K (F), p-Akt/Akt (G), and VEGFA (H), normalized to their respective total protein or loading control (GAPDH). ns, not significant; *P < 0.05, **P < 0.01

To investigate the mechanism by which DPSC-Exos alleviate hair growth inhibition in AGA mice, dorsal skin tissues were randomly collected from three mice each in the MODEL and EXO groups for transcriptome sequencing. Heatmap and volcano plot analysis revealed significant differential gene expression between the two groups (Fig. 7A, B). GO enrichment analysis indicated that the differentially expressed genes were significantly associated with terms such as “vasculature development” and “blood vessel development” (Fig. 7C). KEGG pathway enrichment analysis further demonstrated a marked upregulation of the Pi3k/Akt signaling pathway in the EXO group compared with the MODEL group (Fig. 7D). To validate these findings at the protein level, dorsal skin samples from all four groups (CON, MODEL, EXO, and POSITIVE) were subjected to Western blotting analysis. Compared with the CON group, the MODEL group showed downregulated levels of phosphorylated PI3K (p-PI3K)/total PI3K, phosphorylated Akt (p-Akt)/total Akt, and VEGFA. In contrast, both the EXO and POSITIVE groups exhibited significant upregulation of p-PI3K, p-Akt, and VEGFA relative to the MODEL group (Fig. 7E–H). These results support that DPSC-Exos promote hair regeneration, at least in part, by activating PI3K/Akt signaling and enhancing VEGFA-associated vascular remodeling.

### DPSC-Exos restore pro-angiogenic factor expression and activate PI3K/Akt signaling in DPCs in vitro

**Fig. 8:**
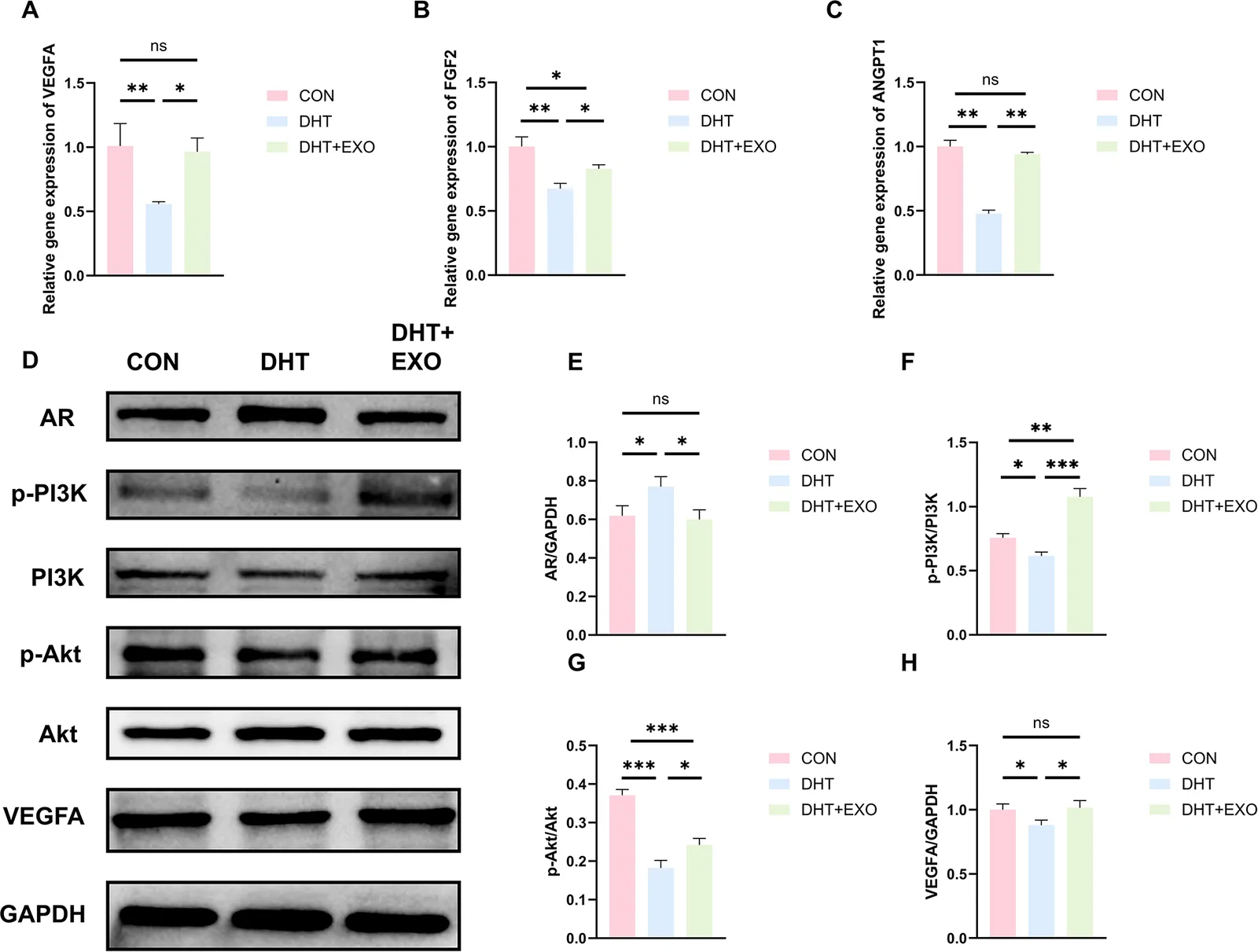
DPSC-Exos restore pro-angiogenic factor expression and activate PI3K/Akt signaling in DPCs in vitro. **A–C** RT-qPCR analysis of pro-angiogenic genes *VEGFA* (**A**), *FGF2* (**B**), and *ANGPT1* (**C**) in DPCs treated as indicated. Gene expression was normalized to *GAPDH*. ns, not significant; *P < 0.05, **P < 0.01. **D** Representative Western blot images showing the expression levels of AR, p-PI3K, total PI3K, p-Akt, total Akt, and VEGFA in DPC lysates. **E–H** Densitometric quantification of protein levels for AR (**E**), p-PI3K/PI3K (**F**), p-Akt/Akt (**G**), and VEGFA (**H**), normalized to their respective total protein or loading control (GAPDH). ns, not significant; *P < 0.05, **P < 0.01, ***P<0.001

To further validate the mechanism at the cellular level, RT-qPCR analysis showed that DHT treatment significantly downregulated the mRNA levels of *VEGFA*, *FGF2*, and *ANGPT1* in DPCs, while DPSC-Exos treatment reversed this Reduction (Fig. 8A–C), indicating that exosomes restore the expression of key pro-angiogenic genes. Western blot analysis showed that DHT treatment significantly upregulated AR expression in DPCs, an effect that was effectively reversed by subsequent treatment with DPSC-Exos. Moreover, compared with the control group, DHT-treated cells exhibited decreased levels of p-PI3K/total PI3K, p-Akt/total Akt, VEGFA. In contrast, DPSC-Exos treatment significantly elevated the expression of these proteins (Fig. 8D–H). These results suggest that DPSC-Exos counteract DHT-induced dysfunction in DPCs, at least in part, by reactivating PI3K/Akt signaling and restoring VEGFA-associated pro-angiogenic programs.

### Pharmacological inhibition of PI3K/Akt abrogates DPSC-Exos–mediated pro-angiogenic and hair-inductive effects

**Fig. 9:**
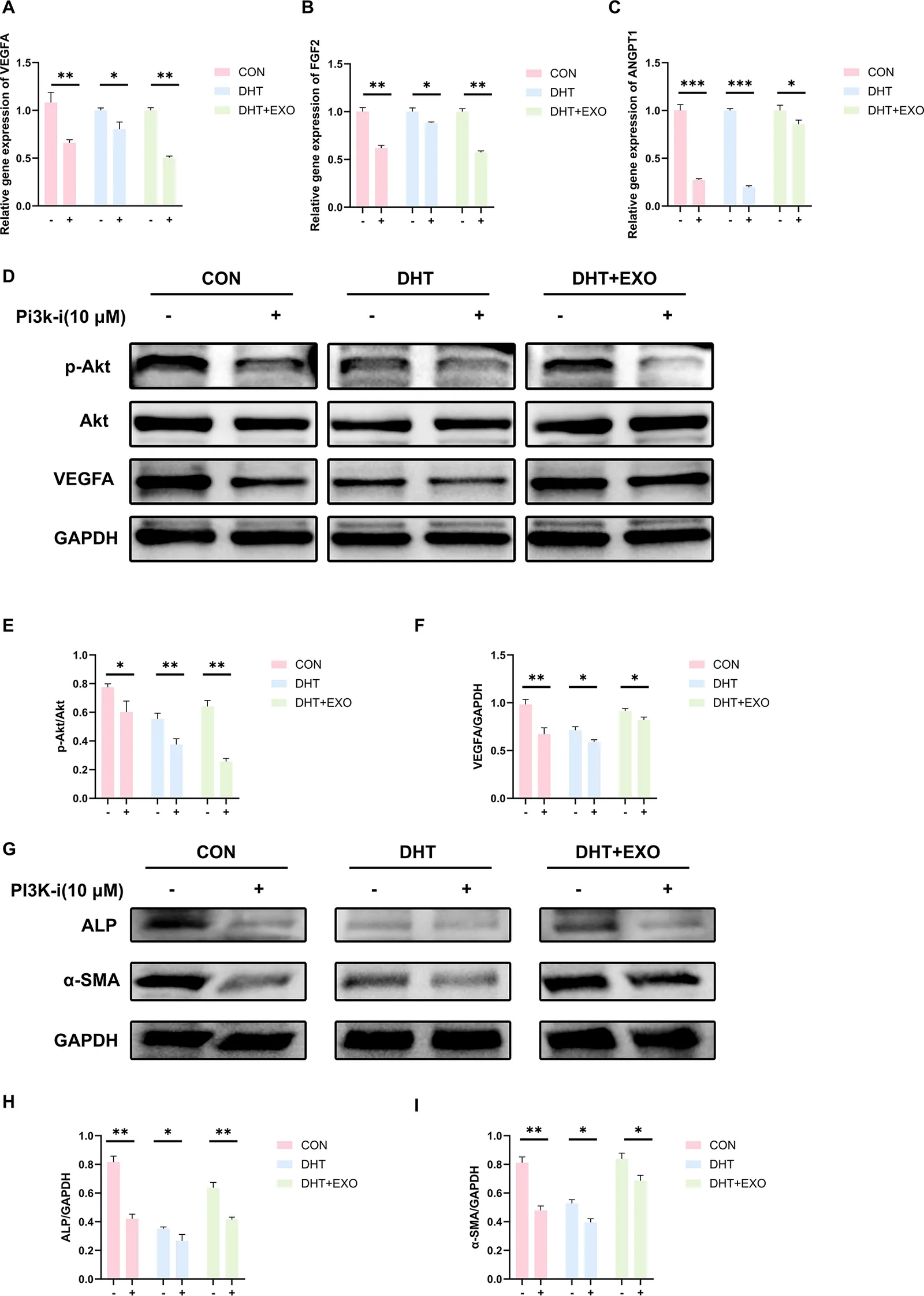
Inhibition of the PI3K/Akt pathway abrogates the pro-angiogenic and hair-inductive effects of DPSC-Exos. **A–C** RT-qPCR analysis of *VEGFA* (**A**), *FGF2* (**B**), and *ANGPT1* (**C**) mRNA levels in DPCs treated as indicated. Gene expression was normalized to *GAPDH*. ns, not significant; *P < 0.05, **P < 0.01, ***P<0.001. **D** Representative Western blot images showing the expression levels of p-Akt, total Akt, and VEGFA. **E**, **F** Densitometric quantification of protein levels for p-Akt/Akt (**E**), and VEGFA (**F**), normalized to their respective total protein or loading control (GAPDH). ns, not significant; *P < 0.05, **P < 0.01. **G** Representative Western blot images showing the expression of hair-inductive markers ALP and α-SMA. **H**,** I** Densitometric quantification of ALP (**H**) and α-SMA (**I**) protein levels. ns, not significant; *P < 0.05, **P < 0.01, ***P<0.001

To confirm the essential role of the PI3K/Akt pathway in DPSC-Exos-mediated effects, a PI3K inhibitor (LY-294002, 10 µM) was applied to DPCs in the following groups: CON, DHT, and DHT + DPSC-Exos (DHT + EXO). As shown by RT-qPCR, the addition of LY-294,002 significantly downregulated the mRNA levels of the pro-angiogenic genes *VEGFA*, *FGF2*, and *ANGPT1* in all three groups (Fig. 9A–C).

Consistently, Western blot analysis revealed that PI3K inhibition markedly reduced the protein levels of VEGFA and p-Akt/total Akt across all treatment conditions (Fig. 9D–F). Furthermore, the expression of the hair-inductive markers ALP and α-SMA was also substantially decreased upon LY-294,002 treatment (Fig. 9G–I). Collectively, these data demonstrate that blocking PI3K/Akt signaling effectively suppresses both the angiogenic and hair-inductive activities induced by DPSC-Exos, confirming the pathway’s necessity for the observed functional recovery.

## Discussion

AGA represents one of the most prevalent forms of hair loss [18]. However, current mainstream therapies such as minoxidil and finasteride exhibit limitations in efficacy and are associated with certain side effects, underscoring the urgent clinical need to explore novel treatment strategies [19, 20]. This study demonstrates for the first time that DPSC-Exos can effectively reverse DHT-induced hair loss by upregulating pro-angiogenic factors (VEGFA, FGF2, ANGPT1) and activating the PI3K/Akt signaling pathway, thereby promoting functional recovery of DPCs and reconstruction of the perifollicular vascular network. In animal models, the therapeutic effect of DPSC-Exos was comparable to that of minoxidil.

Normal hair growth follows a continuous cyclical process comprising the anagen (growth), catagen (regression), and telogen (resting) phases [21]. The precise etiology and pathogenesis of AGA remain incompletely understood, although genetic predisposition, elevated local androgen levels, and overexpression of the AR are widely recognized as major contributing factors [22]. Testosterone is converted by 5α-reductase into the more potent DHT, which subsequently binds to AR [23].

In AGA, the duration of the anagen phase is shortened while the telogen phase is prolonged, ultimately leading to hair loss. Emerging evidence highlights the perifollicular vascular microenvironment as an indispensable unit for sustaining the anagen phase by supplying oxygen, nutrients, and essential growth factors via adequate blood perfusion [24, 25]. Conversely, vascular regression or dysfunction directly contributes to follicular miniaturization and a truncated growth phase [26]. Several existing therapies, such as minoxidil, platelet-rich plasma, and microneedling, aim to ameliorate AGA by improving follicular vascular supply and promoting vasodilation [27–30]. DPSC-Exos have shown considerable promise in regenerative medicine and tissue engineering, particularly in bone regeneration, neuroprotection, and anti-inflammatory applications [31–33]. Previous studies indicate that DPSC-Exos can enhance wound healing by accelerating vascularization [16]. Consistent with these findings, our results demonstrate that DPSC-Exos significantly upregulate key pro-angiogenic factors and promote neovascularization in both in vitro and in vivo settings, thereby establishing a favorable local microenvironment for hair regeneration.

Transcriptomic sequencing and bioinformatic analysis further revealed that DPSC-Exos treatment significantly enriched biological processes related to vascular development and activated the PI3K/Akt signaling pathway. A tightly coupled bidirectional positive feedback regulatory mechanism exists between VEGFA and the PI3K/Akt pathway: VEGFA activates PI3K/Akt signaling via its receptor VEGFR2, thereby promoting endothelial cell survival, migration, and angiogenesis [34–36]. Simultaneously, activated Akt enhances the activity of transcription factors, leading to further upregulation of VEGFA expression and establishing a self-amplifying pro-angiogenic signaling circuit [37, 38]. In this study, DPSC-Exos concurrently elevated levels of p-PI3K and p-Akt while markedly enhancing VEGFA expression. Importantly, the use of the PI3K inhibitor LY-294,002 completely abrogated both the upregulation of VEGFA and its downstream pro-regenerative effects. These results confirm that activation of the PI3K/Akt pathway is a necessary mechanistic step for DPSC-Exos to exert its pro-angiogenic and hair-inductive functions.

In summary, this study elucidates that DPSC-Exos can systematically promote the reconstruction of the perifollicular vascular network and the functional recovery of dermal papilla cells by activating the PI3K/Akt–VEGFA positive feedback circuit, thereby providing a novel cell-free therapeutic strategy with translational potential for AGA treatment. Future research may focus on identifying the specific active components within DPSC-Exos responsible for these effects and exploring their synergistic potential in combination with existing therapies.

## Conclusion

This study demonstrates that DPSC-Exos attenuate DHT-induced hair growth impairment and promote hair regeneration by enhancing perifollicular pro-angiogenic programs and restoring DPC function. Activation of PI3K/Akt signaling and induction of VEGFA-associated pathways are required for these effects, as supported by pharmacological inhibition experiments. Collectively, DPSC-Exos represent a potential cell-free therapeutic modality for AGA and warrant further investigation to advance translational development.

## Supplementary Information

Below is the link to the electronic supplementary material.

Supplementary Material 1.

## Data Availability

No datasets were generated or analysed during the current study.

## Abbreviations

α-SMA: α-Smooth Muscle Actin
AGA: Androgenetic Alopecia
Akt: Protein Kinase B (PKB)
ALP: Alkaline Phosphatase
ANGPT1: Angiopoietin-1
AR: Androgen Receptor
DHT: Dihydrotestosterone
DPC: Dermal Papilla Cell
DPSC: Dental Pulp Stem Cell
DPSC-Exos: DPSC-derived exosomes
FGF2: Fibroblast Growth Factor 2
PI3K: Phosphatidylinositol 3-Kinase
VEGFA: Vascular Endothelial Growth Factor A

## Declarations

### Use of AI and AI-assisted technologies

The authors declare that they have not used artificial intelligence (AI)-generated work in this manuscript.

## References

[1] Chen S, Xie X, Zhang G, et al. Comorbidities in Androgenetic Alopecia: a Comprehensive Review. Dermatol Ther (Heidelb). 2022;12(10):2233–47. 10.1007/s13555-022-00799-7. 36115913 PMC9483356

[2] Fu D, Huang J, Li K, et al. Dihydrotestosterone-induced hair regrowth inhibition by activating androgen receptor in C57BL6 mice simulates androgenetic alopecia. Biomed Pharmacother. 2021;137:111247. 10.1016/j.biopha.2021.111247. 33517191

[3] Nestor MS, Ablon G, Gade A, et al. Treatment options for androgenetic alopecia: efficacy, side effects, compliance, financial considerations, and ethics. J Cosmet Dermatol. 2021;20(12):3759–81. 10.1111/jocd.14537. 34741573 PMC9298335

[4] Dhurat R, Sharma A, Rudnicka L, et al. 5-Alpha reductase inhibitors in androgenetic alopecia: shifting paradigms, current concepts, comparative efficacy, and safety. Dermatol Ther. 2020;33(3):e13379. 10.1111/dth.13379. 32279398

[5] York K, Meah N, Bhoyrul B, et al. A review of the treatment of male pattern hair loss. Expert Opin Pharmacother. 2020;21(5):603–12. 10.1080/14656566.2020.1721463. 32066284

[6] Devjani S, Ezemma O, Kelley KJ, et al. Androgenetic Alopecia: therapy Update. Drugs. 2023;83(8):701–15. 10.1007/s40265-023-01880-x. 37166619 PMC10173235

[7] Matsuzaka Y, Yashiro R. Therapeutic Strategy of Mesenchymal-Stem-Cell-Derived Extracellular Vesicles as Regenerative Medicine. Int J Mol Sci. 2022. 10.3390/ijms23126480. 35742923 PMC9224400

[8] Keshtkar S, Azarpira N, Ghahremani MH. Mesenchymal stem cell-derived extracellular vesicles: novel frontiers in regenerative medicine. Stem Cell Res Ther. 2018;9(1):63. 10.1186/s13287-018-0791-7. 29523213 PMC5845209

[9] Kalluri R, LeBleu VS. The biology, function, and biomedical applications of exosomes. Science. 2020. 10.1126/science.aau6977. 32029601 PMC7717626

[10] Gurunathan S, Kang MH, Kim JH. A Comprehensive Review on Factors Influences Biogenesis, Functions, Therapeutic and Clinical Implications of Exosomes. Int J Nanomed. 2021;16:1281–312. 10.2147/ijn.S291956. PMC7898217 33628021

[11] Yousefian F, Espinoza L, Yadlapati S, et al. A comprehensive review of the medical and cosmetic applications of exosomes in dermatology. J Cosmet Dermatol. 2024;23(4):1224–8. 10.1111/jocd.16149. 38226413

[12] Yu H, Feng H, Zeng H, et al. Exosomes: the emerging mechanisms and potential clinical applications in dermatology. Int J Biol Sci. 2024;20(5):1778–95. 10.7150/ijbs.92897. 38481799 PMC10929203

[13] Contessi Negrini N, Angelova Volponi A, Higgins CA, et al. Scaffold-based developmental tissue engineering strategies for ectodermal organ regeneration. Mater Today Bio. 2021;10:100107. 10.1016/j.mtbio.2021.100107. 33889838 PMC8050778

[14] Reynolds AJ, Jahoda CA. Cultured human and rat tooth papilla cells induce hair follicle regeneration and fiber growth. Differentiation. 2004;72(9–10):566–75. 10.1111/j.1432-0436.2004.07209010.x 15617567

[15] Chen H, Yamaguchi S, Wang Y, et al. Cytoprotective role of human dental pulp stem cell-conditioned medium in chemotherapy-induced alopecia. Stem Cell Res Ther. 2024;15(1):84. 10.1186/s13287-024-03695-3. 38500206 PMC10949570

[16] Zhou Z, Zheng J, Lin D, et al. Exosomes derived from dental pulp stem cells accelerate cutaneous wound healing by enhancing angiogenesis via the Cdc42/p38 MAPK pathway. Int J Mol Med. 2022. 10.3892/ijmm.2022.5199. 36321793 PMC9662140

[17] Mao Y, Liu P, Wei J, et al. Exosomes derived from Umbilical cord mesenchymal stem cell promote hair regrowth in C57BL6 mice through upregulation of the RAS/ERK signaling pathway. J Transl Int Med. 2024;12(5):478–94. 10.1515/jtim-2024-0012. 39513036 PMC11538887

[18] Griggs J, Burroway B, Tosti A. Pediatric androgenetic alopecia: a review. J Am Acad Dermatol. 2021;85(5):1267–73. 10.1016/j.jaad.2019.08.018. 31415838

[19] Gupta AK, Talukder M, Venkataraman M, et al. Minoxidil: a comprehensive review. J Dermatolog Treat. 2022;33(4):1896–906. 10.1080/09546634.2021.1945527. 34159872

[20] Gupta AK, Talukder M, Williams G. Comparison of oral minoxidil, finasteride, and dutasteride for treating androgenetic alopecia. J Dermatolog Treat. 2022;33(7):2946–62. 10.1080/09546634.2022.2109567. 35920739

[21] Santos Z, Avci P, Hamblin MR. Drug discovery for alopecia: gone today, hair tomorrow. Expert Opin Drug Discov. 2015;10(3):269–92. 10.1517/17460441.2015.1009892. 25662177 PMC4339524

[22] Xiao Y, Zhang Y, Deng S, et al. Immune and Non-immune Interactions in the Pathogenesis of Androgenetic Alopecia. Clin Rev Allergy Immunol. 2025;68(1):22. 10.1007/s12016-025-09034-5. 40024940

[23] Yun SI, Lee SK, Goh EA, et al. Weekly treatment with SAMiRNA targeting the androgen receptor ameliorates androgenetic alopecia. Sci Rep. 2022;12(1):1607. 10.1038/s41598-022-05544-w. 35102171 PMC8803970

[24] Zhou S, Li Z, Li X, et al. Crosstalk between endothelial cells and dermal papilla entails hair regeneration and angiogenesis during aging. J Adv Res. 2025;70:339–53. 10.1016/j.jare.2024.05.006. 38718895 PMC11976415

[25] Abaci HE, Coffman A, Doucet Y, et al. Tissue engineering of human hair follicles using a biomimetic developmental approach. Nat Commun. 2018;9(1):5301. 10.1038/s41467-018-07579-y. 30546011 PMC6294003

[26] Yoshida Y, Soma T, Kishimoto J. Characterization of human dermal sheath cells reveals CD36-expressing perivascular cells associated with capillary blood vessel formation in hair follicles. Biochem Biophys Res Commun. 2019;516(3):945–50. 10.1016/j.bbrc.2019.06.146. 31272715

[27] Yuan A, Xia F, Bian Q, et al. Ceria Nanozyme-Integrated Microneedles Reshape the Perifollicular Microenvironment for Androgenetic Alopecia Treatment. ACS Nano. 2021;15(8):13759–69. 10.1021/acsnano.1c05272. 34279913

[28] Ding YW, Li Y, Zhang ZW, et al. Hydrogel forming microneedles loaded with VEGF and Ritlecitinib/polyhydroxyalkanoates nanoparticles for mini-invasive androgenetic alopecia treatment. Bioact Mater. 2024;38:95–108. 10.1016/j.bioactmat.2024.04.020. 38699241 PMC11061199

[29] Cruciani M, Masiello F, Pati I, et al. Platelet-rich plasma for the treatment of alopecia: a systematic review and meta-analysis. Blood Transfus. 2023;21(1):24–36. 10.2450/2021.0216-21. 34967722 PMC9918380

[30] Goren A, Naccarato T. Minoxidil in the treatment of androgenetic alopecia. Dermatol Ther. 2018;31(5):e12686. 10.1111/dth.12686. 30155952

[31] Lee AE, Choi JG, Shi SH, et al. DPSC-Derived Extracellular Vesicles Promote Rat Jawbone Regeneration. J Dent Res. 2023;102(3):313–21. 10.1177/00220345221133716. 36348514

[32] Mondal K, Ghanty R, Mahadevan A, et al. Intranasal delivery of DPSC-derived small extracellular vesicles-encased phloroglucinol attenuates non-motor and motor deficits and promotes neurogenesis in an in vivo rat model of Parkinson’s disease. Stem Cell Res Ther. 2025;16(1):570. 10.1186/s13287-025-04573-2. 41102844 PMC12532972

[33] Qiao X, Tang J, Dou L, et al. Dental Pulp Stem Cell-Derived Exosomes Regulate Anti-Inflammatory and Osteogenesis in Periodontal Ligament Stem Cells and Promote the Repair of Experimental Periodontitis in Rats. Int J Nanomed. 2023;18:4683–703. 10.2147/ijn.S420967. PMC10441659 37608819

[34] Liu X, He H, Zhang F, et al. m6A methylated EphA2 and VEGFA through IGF2BP2/3 regulation promotes vasculogenic mimicry in colorectal cancer via PI3K/AKT and ERK1/2 signaling. Cell Death Dis. 2022;13(5):483. 10.1038/s41419-022-04950-2. 35595748 PMC9122982

[35] Liu L, Gao Y, Yao S. Transthyretin-Regulated Diabetic Retinopathy Through the VEGFA/PI3K/AKT Pathway. Invest Ophthalmol Vis Sci. 2024;65(1):45. 10.1167/iovs.65.1.45. 38289614 PMC10833055

[36] Chen L, Zhang H, Zhang Y, et al. Ganglion cell-derived LysoPS induces retinal neovascularisation by activating the microglial GPR34-PI3K-AKT-NINJ1 axis. J Neuroinflammation. 2024;21(1):278. 10.1186/s12974-024-03265-7. 39468551 PMC11520652

[37] Zhu GS, Tang LY, Lv DL, et al. Total Flavones of Abelmoschus manihot Exhibits Pro-Angiogenic Activity by Activating the VEGF-A/VEGFR2-PI3K/Akt Signaling Axis. Am J Chin Med. 2018;46(3):567–83. 10.1142/s0192415x18500295. 29595071

[38] Li X, Yang W, Dai C, et al. Integrative multi-Omics and network pharmacology reveal angiogenesis promotion by Quan-Du-Zhong Capsule via VEGFA/PI3K-Akt pathway. J Ethnopharmacol. 2025;340:119222. 10.1016/j.jep.2024.119222. 39647590

