# Supplementary material for: Dental pulp stem cell exosomes promote angiogenesis via the PI3K/Akt signaling pathway to treat androgenetic alopecia
Source: Stem Cell Res Ther. 2026 May 21;17:325. doi: 10.1186/s13287-026-05056-8 (PMC13587414; doi:10.1186/s13287-026-05056-8)
Supplement: Supplementary file 1 [file 13287_2026_5056_MOESM1_ESM.pdf]

Fig. 3C

LANE 1-CON
LANE 2-EXO

α-SMA

GAPDH

ALP

GAPDH

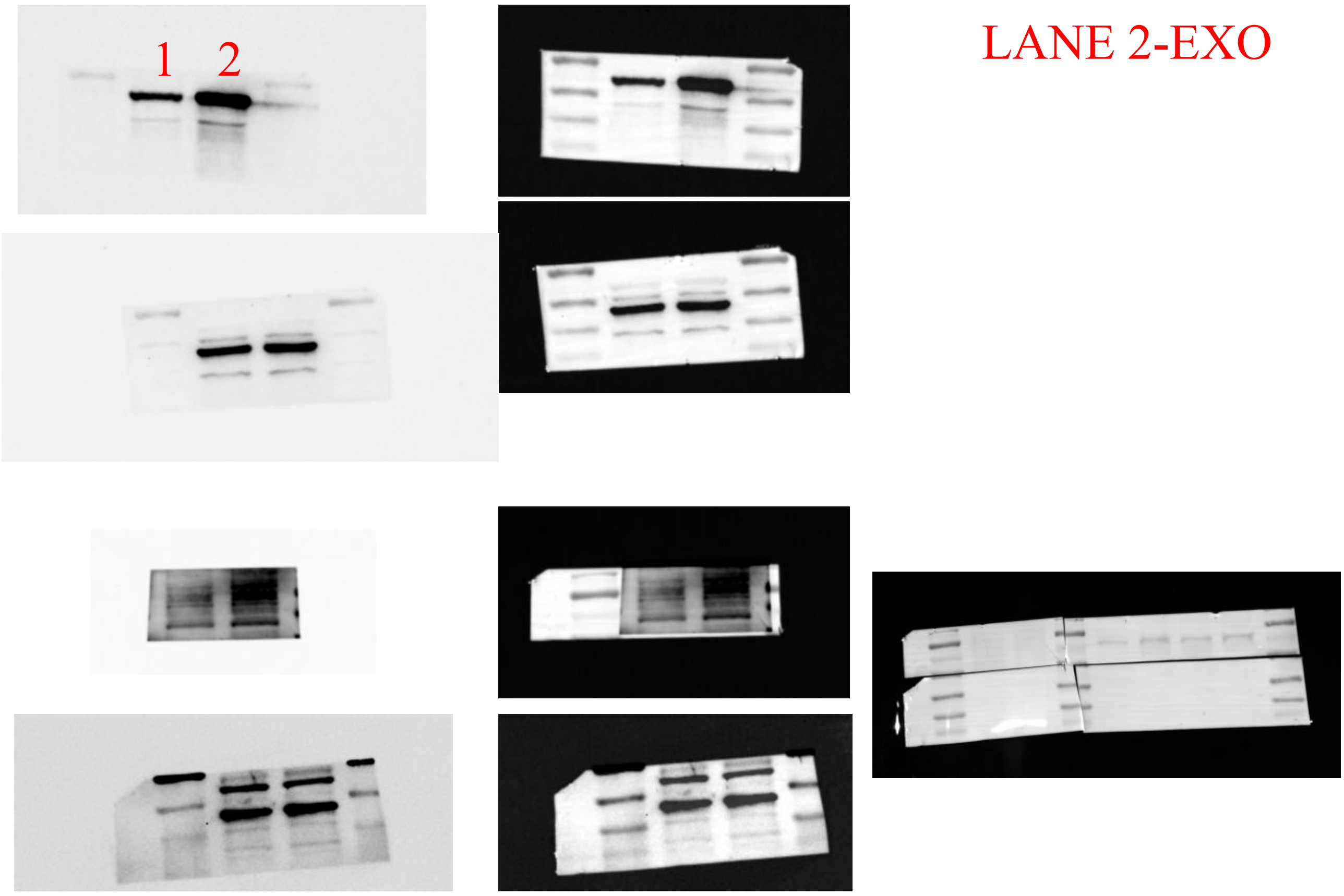

Fig. 4E

LANE 1-CON
LANE 2-DHT
LANE 3-DHT+EXO

ALP

α-SMA

GAPDH

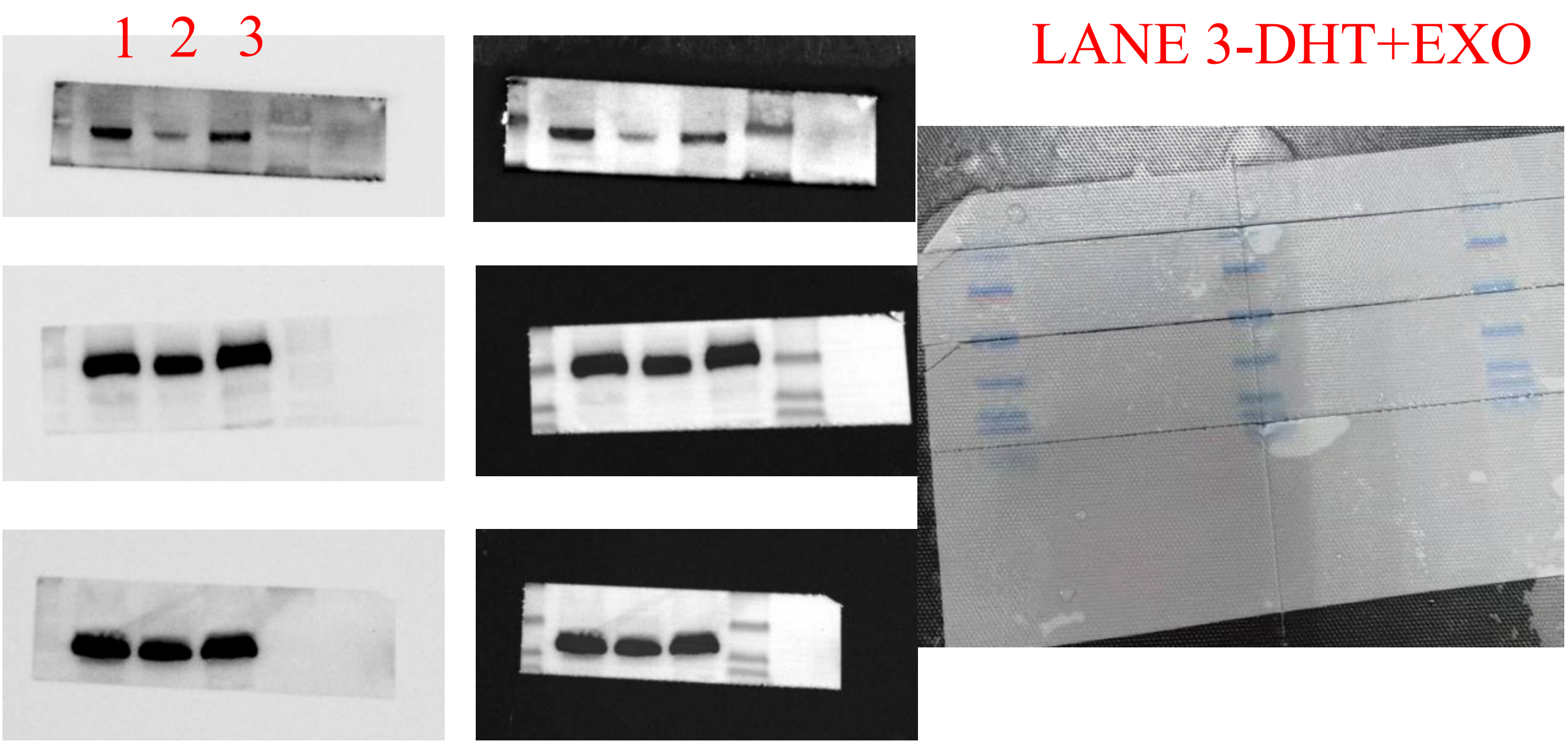

Fig. 6D

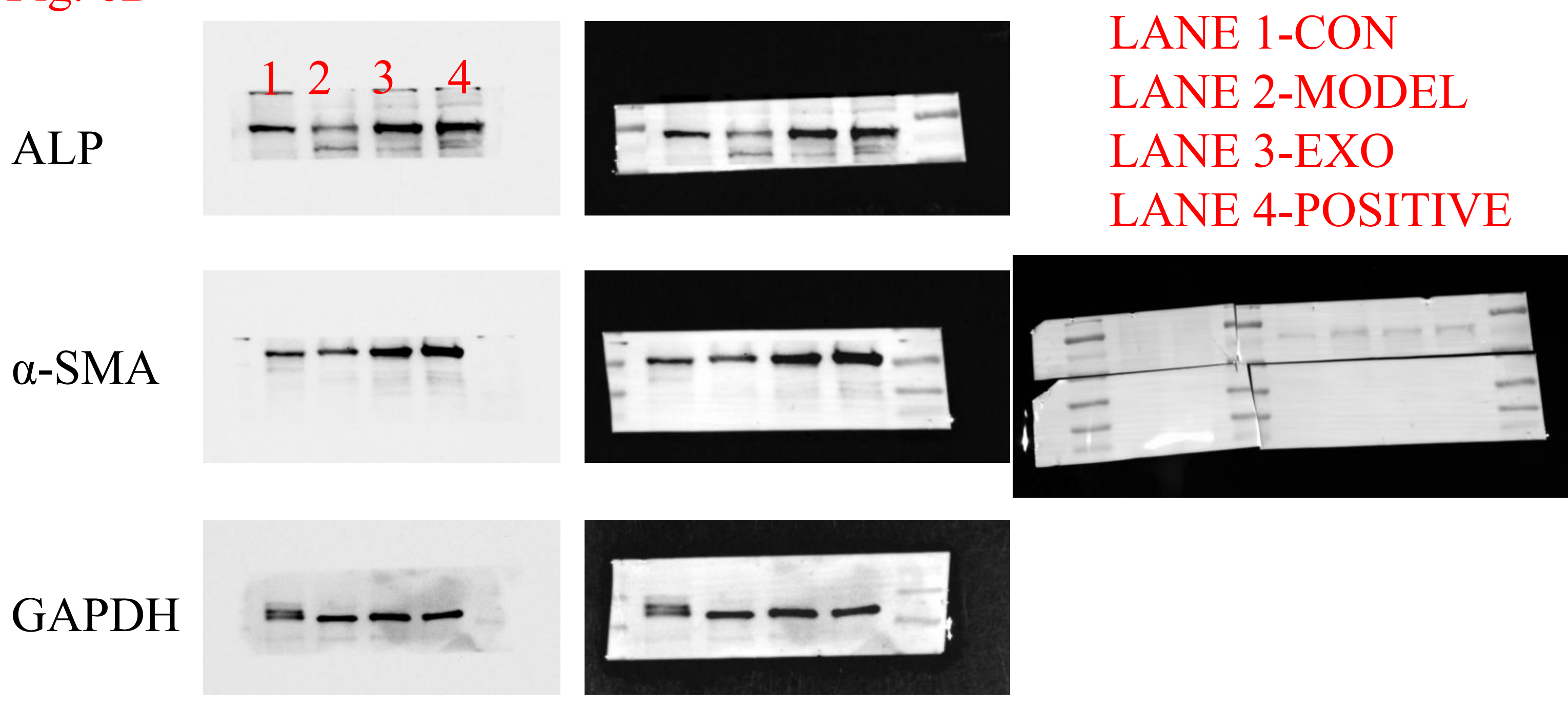
1 2 3 4
ALP
α-SMA
GAPDH
LANE 1-CON
LANE 2-MODEL
LANE 3-EXO
LANE 4-POSITIVE

Fig. 7E

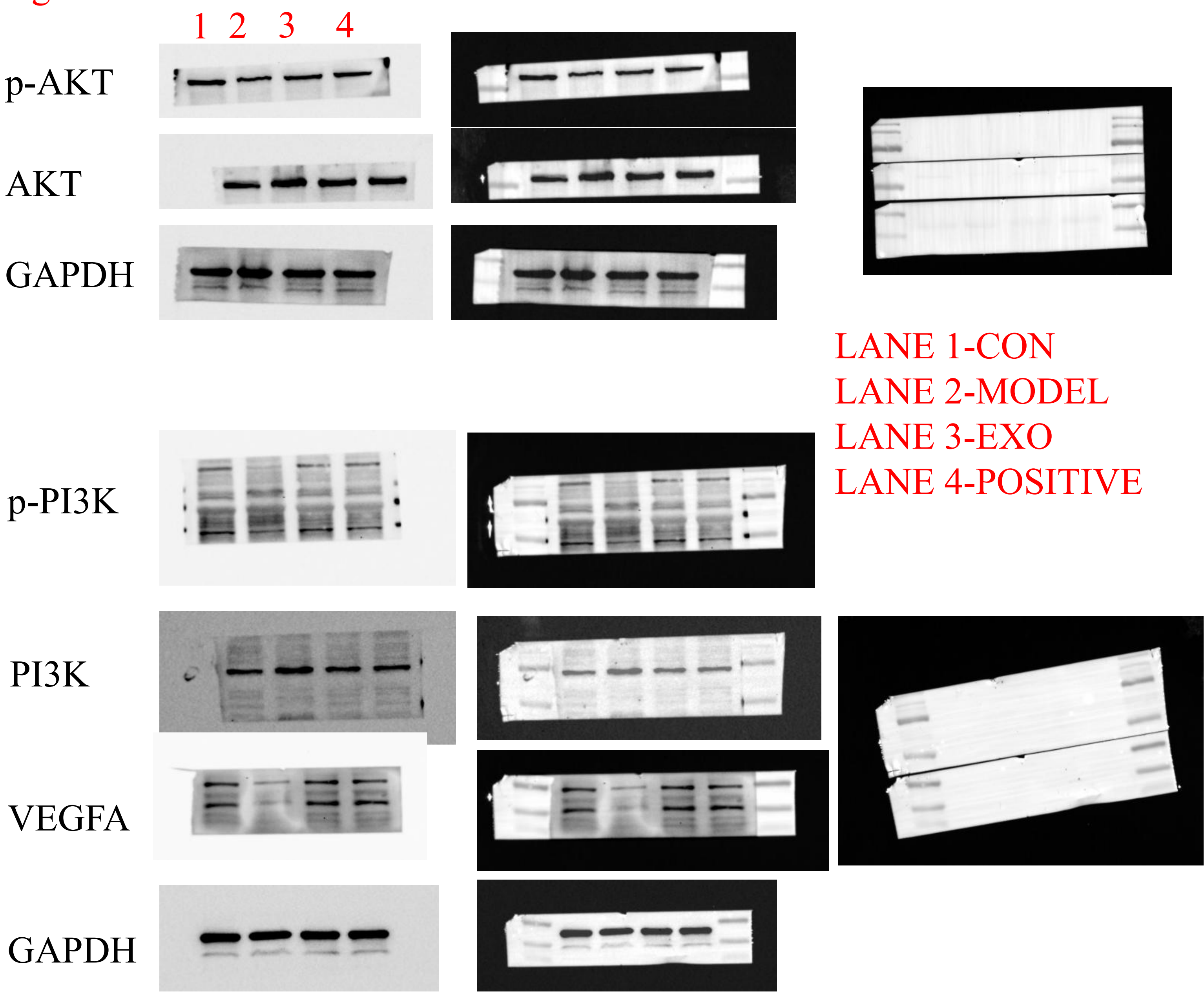
1 2 3 4
p-AKT
AKT
GAPDH
LANE 1-CON
LANE 2-MODEL
LANE 3-EXO
LANE 4-POSITIVE
p-PI3K
PI3K
VEGFA
GAPDH

Fig. 8D

p-AKT

AKT

VEGFA

GAPDH

AR

PI3K

GAPDH

p-PI3K

GAPDH

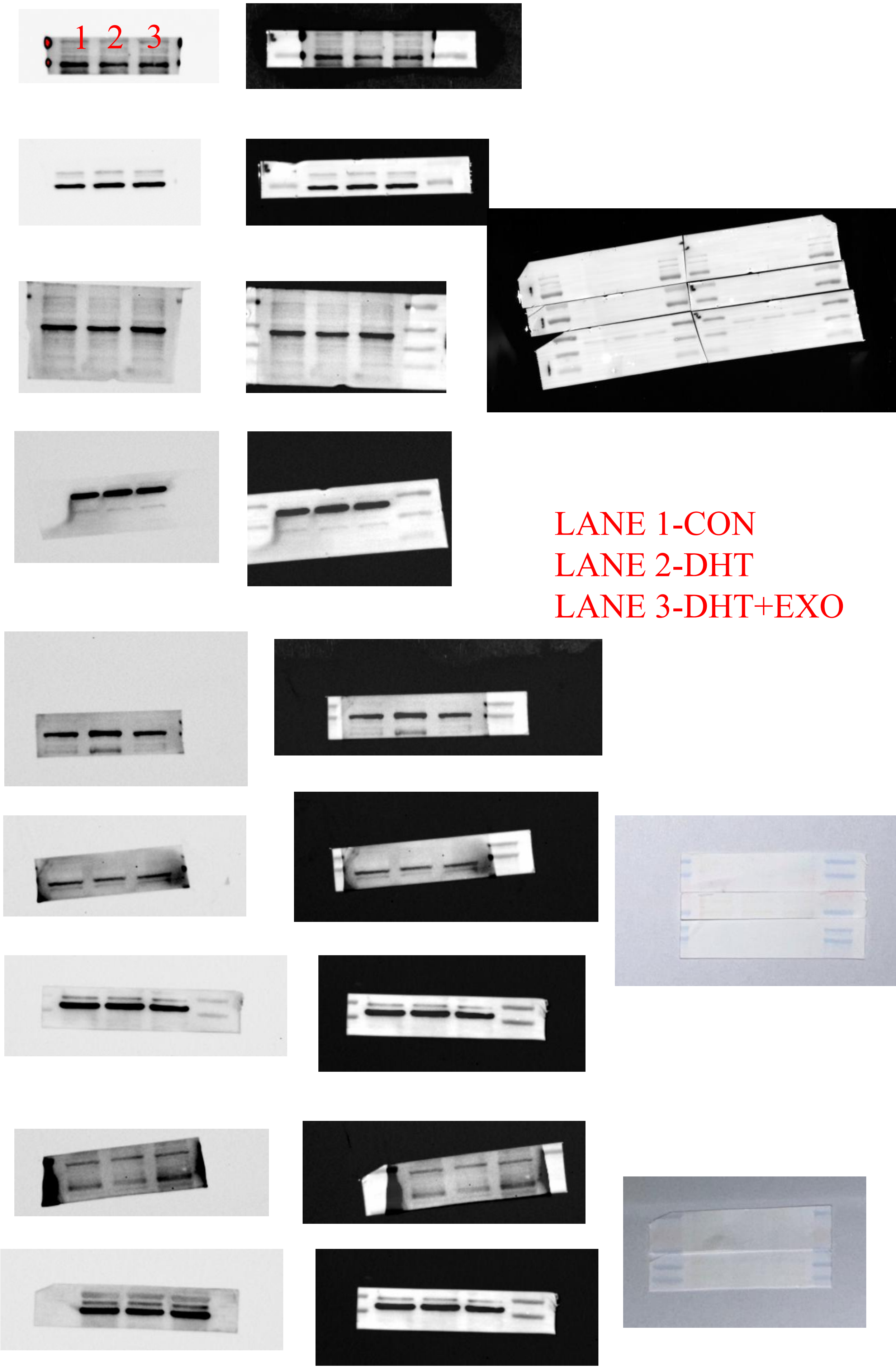

Fig. 9D

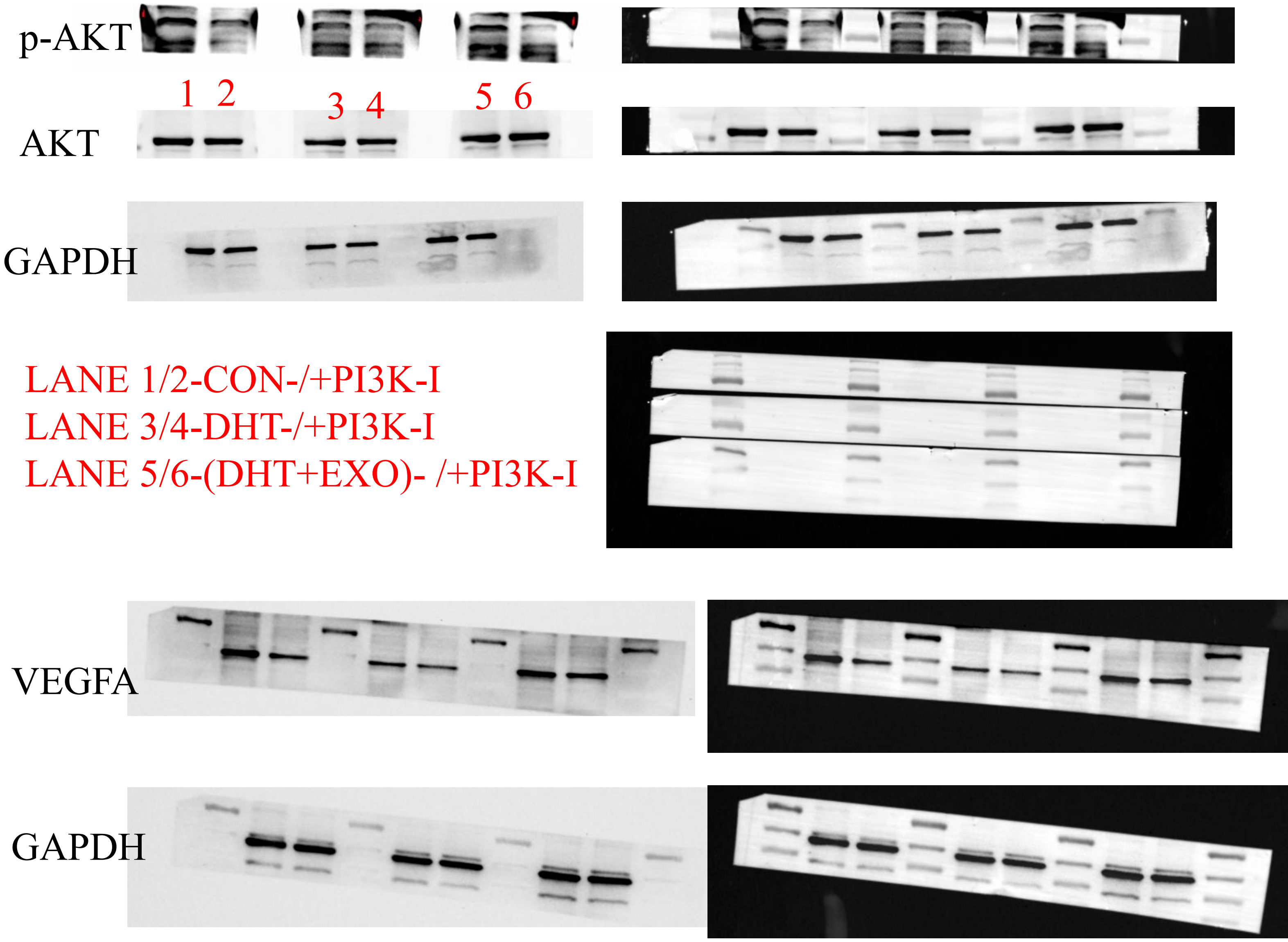
p-AKT
1 2
3 4
5 6
AKT
GAPDH
LANE 1/2-CON-/+PI3K-I
LANE 3/4-DHT-/+PI3K-I
LANE 5/6-(DHT+EXO)- /+PI3K-I
VEGFA
GAPDH

Fig. 9G

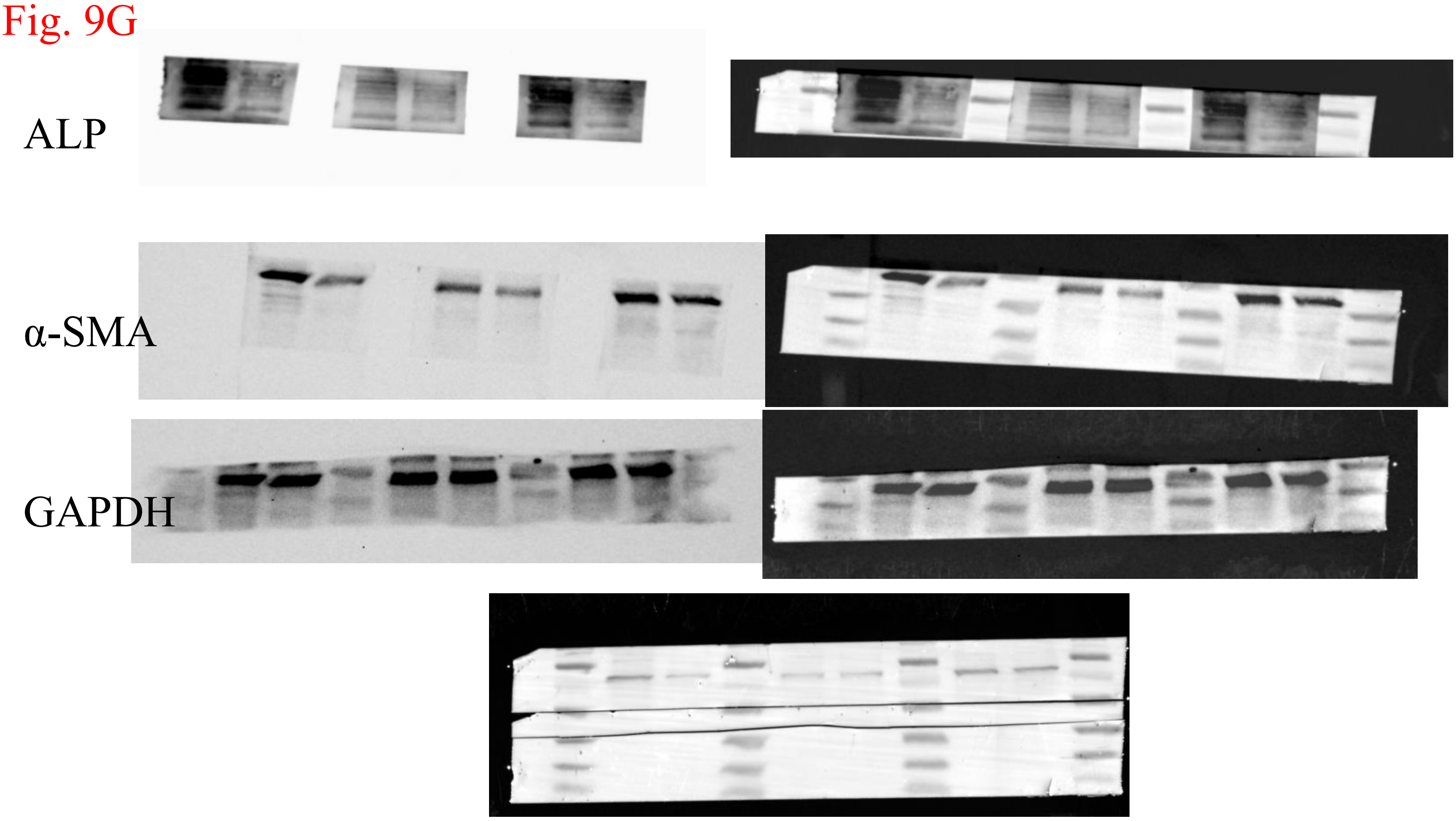
ALP
α-SMA
GAPDH
